# Advances in understanding the mechanism of resistance to anthracnose and induced defence response in tea plants

**DOI:** 10.1111/mpp.13354

**Published:** 2023-07-31

**Authors:** Anburaj Jeyaraj, Tamilselvi Elango, Xuan Chen, Jing Zhuang, Yuhua Wang, Xinghui Li

**Affiliations:** ^1^ College of Horticulture Nanjing Agricultural University Nanjing China

**Keywords:** anthracnose, *Camellia sinensis*, *Colletotrichum*, host–pathogen interaction, plant resistance

## Abstract

The tea plant (*Camellia sinensis*) is susceptible to anthracnose disease that causes considerable crop loss and affects the yield and quality of tea. Multiple *Colletotrichum* spp. are the causative agents of this disease, which spreads quickly in warm and humid climates. During plant–pathogen interactions, resistant cultivars defend themselves against the hemibiotrophic pathogen by activating defence signalling pathways, whereas the pathogen suppresses plant defences in susceptible varieties. Various fungicides have been used to control this disease on susceptible plants, but these fungicide residues are dangerous to human health and cause fungicide resistance in pathogens. The problem‐solving approaches to date are the development of resistant cultivars and ecofriendly biocontrol strategies to achieve sustainable tea cultivation and production. Understanding the infection stages of *Colletotrichum*, tea plant resistance mechanisms, and induced plant defence against *Colletotrichum* is essential to support sustainable disease management practices in the field. This review therefore summarizes the current knowledge of the identified causative agent of tea plant anthracnose, the infection strategies and pathogenicity of *C. gloeosporioides*, anthracnose disease resistance mechanisms, and the caffeine‐induced defence response against *Colletotrichum* infection. The information reported in this review will advance our understanding of host–pathogen interactions and eventually help us to develop new disease control strategies.

## Graphical abstract

This review summarizes the causative agent of tea anthracnose, infection strategies and pathogenicity of *C. gloeosporioides*, host–pathogen interactions, plant resistance mechanisms, and induced defence against *Colletotrichum*.

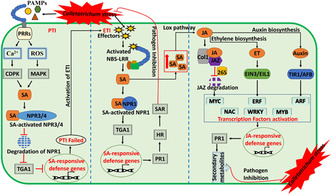

## 1 INTRODUCTION

Tea (*Camellia sinensis*), a plant belonging to the family *Theaceae*, is an economically important woody perennial plantation crop that is widespread throughout tropical and subtropical areas such as China, India, Kenya, and Sri Lanka (Mukhopadhyay et al., 2016). The tender shoots of tea plants are used to produce the popular nonalcoholic beverage known as tea due to the abundant production of many valuable secondary metabolites, such as polyphenols, catechins, flavanones, caffeine, theanine, saponin, vitamins, minerals and volatile oils (Jeyaraj et al., 2014). As an evergreen woody plant, the growth of tea plants and quality of teas is susceptible to various biotic stresses (bacterial, fungal, and viral diseases) under natural conditions, which affect normal growth and development, and cause a great loss in tea yield and quality worldwide (Jeyaraj et al., 2019). Anthracnose is an economically devastating leaf disease in tea plants. Pathogens of tea plant anthracnose, multiple *Colletotrichum* spp., a large genus of Ascomycete fungi, which are prevalent under warm and humid conditions and cause severe plant damage (Jeyaraj et al., 2020; Wang, Hao, et al., 2016). Anthracnose is the most common name given to diseases caused by *Colletotrichum* spp. The various *Colletotrichum* species have been reported in the main tea‐growing regions of China and cause several tea diseases known as tea leaf blight, tea brown blight, and tea plant anthracnose (Chen et al., 2017; Guo et al., 2014; Wang, Hao, et al., 2016). As the disease progresses, symptoms of brown blight caused by *Colletotrichum gloeosporioides* on *C. sinensis* began as small, water‐soaked lesions on young leaves and twigs, and later became larger, dark‐brown, necrotic lesions (Guo et al., 2014). Symptoms of tea plant anthracnose caused by *Colletotrichum fructicola* begin as small, water‐soaked lesions on young leaves and later became larger, semicircular or irregular, dark brown and reddish‐brown at the margin. At later stages, lesions became greyish white with black acervuli on the surface (Shi et al., 2018). Recently, Hu et al. (2021) reported that tea leaf blight mainly damages tea plant leaves in three stages: (1) in the early stage, the tips or edges of the diseased leaves initially show yellow‐brown and water‐soaked disease spots; (2) in the middle stage, disease spots then form irregular patches of light brown and grey; and (3) in the later stage, the disease spots display black flat circular spots arranged along with the rosette. Severe damage caused by *Colletotrichum* species can eventually lead to defoliation, affecting both young and old leaves (Fang et al., 2013). It was recently reported that the loss of tea yield induced by *C. fructicola* was estimated to range from 30% to 50% and caused mass defoliation in Guangdong Province, China (Shi et al., 2018).

During plant–fungal interactions, resistant plants defend themselves against pathogen attack by activating intricate immune responses or defence signalling pathways to the particular pathogen, which usually result in a disease‐resistance response (Dobón et al., 2015; Jones & Dangl, 2006). On the other hand, pathogens have developed habits to bypass plant defences, and susceptibility to pathogens reappears; plant susceptibility to pathogens is usually considered from the perspective of the loss of resistance (Gorshkov & Tsers, 2021). The cultivars Longjing 43 (LJ43) and Zhongcha 108 (ZC108) have been used as anthracnose‐susceptible and anthracnose‐resistant tea plant materials, respectively, in several studies to identify the resistance mechanism of tea plants to anthracnose disease (Jeyaraj et al., 2021; Wang et al., 2018; Wang, Hao, et al., 2016). ZC108 has early sprouting time, disease tolerance against *Colletotrichum camelliae*, and high quality green tea compared to LJ43 (Wang, Hao, et al., 2016). Transcriptome analysis in the leaves of both LJ43 and ZC108 revealed that mitogen‐activated protein kinase (MAPK)‐mediated activation of resistance (R) genes are involved in the hypersensitive response (HR) and H_2_O_2_ accumulation in tea plant in response to *C. fructicola* infection (Wang et al., 2018). During the tea–*C. camelliae* interaction, the levels of the precursors and intermediate products of jasmonic acid (JA) and indole‐3‐acetate (IAA) biosynthesis increased in LJ43 (Lu et al., 2020). Recently, Jeyaraj et al. (2021) reported that the susceptibility of LJ43 against *C. gloeosporioides* stress is mainly due to the repression of auxin signal by its co‐repressor and also by increased salicylic acid (SA) synthesis. These phytohormones, SA, JA, and IAA, are mediators of the plant stress response to biotic stresses. However, the effective activation of defence responses to tea plant anthracnose regulated by SA, JA, and IAA‐signalling pathways remains unclear in tea plants.

Tea plants have to be continuously grown in the field for a very long time (>50 years) at the same site (Mukhopadhyay et al., 2016). Tea growers have therefore used various chemical fungicides with different modes of action, which can competently fight against pathogens and control this disease, but these chemical fungicides cause pollution problems affecting the health of humans and animals due to their residual toxicity and also can lead to the development of fungicide resistance in phytopathogenic fungi (Rabha et al., 2014). The problem‐solving approaches are the development of resistant cultivars that typically facilitate the production of high‐yielding, good quality tea plants combined with ecofriendly biocontrol strategies that are promising non‐chemical alternatives to assist plants to fight pathogens (Pandey et al., 2018; Vitale et al., 2020). Existing evidence indicates that the biological role of caffeine is thought to be the strengthening of the plant's defence capabilities (Jeyaraj et al., 2021; Li et al., 2016). The various advanced techniques in bioinformatics and molecular biology for the identification of anthracnose disease‐resistance mechanisms and induced defence in tea plants are powerful approaches for crop improvement (Jeyaraj et al., 2021; Wang, Hao, et al., 2016). Thus, understanding of the infection strategies and pathogenicity of the pathogen, host–pathogen interactions, tea plant resistance mechanisms, and induced plant defence against *Colletotrichum* will provide new control strategies for anthracnose disease prevention and management.

## 2 IDENTIFICATION AND PATHOGENICITY OF *COLLETOTRICHUM* SPP. IN TEA PLANTS

### 2.1 The causative agent of tea plant anthracnose

Tea plant anthracnose disease is highly destructive and has become extremely limiting for tea cultivation and the tea industry (Wang et al., 2018). Diverse *Colletotrichum* species have been isolated and identified from the leaves of tea plants with visible anthracnose symptoms by a single‐spore isolation technique described by Cai et al. (2009). For molecular level confirmation, total genomic DNA isolated from the mycelia of fungi is used as a template for PCR amplification of the ribosomal internal transcribed spacer (ITS), actin (*ACT*), glyceraldehyde‐3‐phosphate dehydrogenase (*GAPDH*), β‐tubulin (*TUB2*), partial sequences of the chitin synthase 1 (*CHS‐1*), calmodulin (*CAL*), glutamine synthetase (*GS*), mating‐type protein, and the Apn2‐Mat1‐2 intergenic spacer (*ApMat*) using gene‐specific forward and reverse primers. The purified PCR products are sequenced for phylogenetic tree construction and analysis of multilocus gene sequences (Wang, Hao, et al., 2016). For morphological confirmation, colony, conidia, conidiophores, and appressoria characteristics are recorded and determined using methods described by Cai et al. (2009). Using multigene molecular phylogenetic analysis coupled with morphological characterization, a total of 10 well‐characterized species (*C. acutatum*, *C. alienum*, *C. boninense*, *C. camelliae*, *C. cliviae*, *C. fioriniae*, *C. fructicola*, *C. gloeosporioides*, *C. karstii*, *C. siamense*), three new record species (*C. aenigma*, *C. endophytica*, *C. truncatum*), four novel species (*C. chongqingense*, *C. henanense*, *C. jiangxiense, C. wuxiense*), and one unidentified strain (*Colletotrichum* sp.) have been found to cause tea plant anthracnose in China (Chen et al., 2017; Guo et al., 2014; Liu et al., 2015; Shi et al., 2018; Wan et al., 2021; Zhang et al., 2021). Of these species, *C. gloeosporioides* complex, *C. camelliae*, and *C. fructicola* were the dominant species causing anthracnose in *C. sinensis* (Zhang et al., 2021). Detailed information about the morphological characterization of the identified *Colletotrichum* species in *C. sinensis* is presented in Table S1. In addition, *C. karstii* isolates grown on potato dextrose agar showed that the upper surface colour of the colonies was pale yellowish to olivaceous with visible salmon‐coloured conidial masses, the conidia were cylindrical, hyaline, aseptate, straight or slightly curved and obtuse at both apexes, and the length and width of conidia varied from 14.1–18.4 μm × 6.8–8.6 μm (Wan et al., 2021). Liu et al. (2015) provided a description of the sexual morphology of *C. cliviae*, which developed on *Anthriscus* stems: ascomata, globose, brown to black, covered by sparse and white aerial mycelium, outer wall composed of flattened angular cells; asci, cylindrical, eight‐spored (62–92 × 8–12 μm); ascospores, uni‐ or biseriately arranged, hyaline, aseptate, smooth‐walled, allantoid, ellipsoidal or ovoid with rounded ends (11–16.5 × 4–6.5 μm). The morphological description and illustration of *C. alienum*, *C. boninense*, *C. fioriniae*, and *C. truncatum* from diseased tea leaves has not been elaborately described yet, but these species were identified based on multilocus phylogenetic analyses (Liu et al., 2015; Wang, Hao, et al., 2016).

Pathogenicity is the potential capacity to cause disease in host plants. Different fungal species display different levels of virulence toward their host plant (Lu et al., 2018). To confirm pathogenicity, Koch's postulates have been conducted in tea plants as described by Cai et al. (2009). In detail, the healthy tea leaves were surface‐sterilized with 75% ethanol and sterile distilled water, and then inoculated with a conidial suspension of *Colletotrichum* isolates (10^5^ conidia mL^−1^) using the wound/drop and nonwound/drop inoculation methods. Leaves inoculated with sterile water were used as control. The inoculated samples were incubated at room temperature in normal light regimes in the greenhouse for 14 days (Liu et al., 2015). Based on the pathogenicity tests, *C. acutatum*, *C. aenigma*, *C. camelliae*, *C. endophytica*, *C. fructicola*, *C. gloeosporioides*, *C. henanense*, and *C. jiangxiense* produce the typical brown lesions of anthracnose disease around wounded areas after 14 days, suggesting these species are more invasive than other species after the inoculation of the leaves of *C. sinensis* (Chen et al., 2017; Guo et al., 2014; Liu et al., 2015; Lu et al., 2018; Shi et al., 2018; Wang, Hao, et al., 2016). It has also been reported that *C. camelliae* and *C. fructicola* are the dominant species causing anthracnose in *C. sinensis*, and *C. camelliae* has higher virulence than *C. fructicola* (Lu et al., 2018; Wang, Hao, et al., 2016).

### 2.2 Infection strategies of *C. gloeosporioides* complex

Tea plant anthracnose disease can be caused by genetically distinct sets of *Colletotrichum* species, including *C. camelliae*, *C. fructicola*, and *C. gloeosporioides*. Multigene molecular phylogenetic analysis revealed that these species belong to the *C. gloeosporioides* species complex (Lu et al., 2018; Wang, Hao, et al., 2016), therefore *C. gloeosporioides* is an excellent model for studying the molecular and cellular bases of fungal pathogenicity in tea plants. Generally, *Colletotrichum* uses a hemibiotrophic infection strategy to invade and colonize host plants; in this strategy, pathogen initially develops biotrophic hyphae inside a living host via appressorial penetration, which later switches to necrotrophic secondary mycelia (Spanu & Panstruga, 2017). Tea plant anthracnose is mostly spread through rain splashes or wind that transport the conidia from necrotic lesions of infected leaves to healthy leaves (Nwankiti et al., 1987). The different stages of infection are attachment and germination of conidia on surface of plants, development of germ tube, differentiation into appressoria and penetration of host cells, development of intracellular hyphae and secondary necrotrophic hyphae, and colonization of plant tissues (Perfect et al., 1999) (Figure S1). The acervuli at the site of infection (lesions) caused by *C. gloeosporioides* release conidia, which are dispersed by rain splashes or wind. After landing a conidium on the surface of leaves, *C. gloeosporioides* can initially infect and penetrate host tissues by a series of specialized infection processes, including the germination of conidia and the formation of appressoria, which penetrate the host cuticle and epidermal cell walls directly (Morin et al., 1996; Perfect et al., 1999) (Figure S1). In addition, Kumar et al. (2001) found that *C. gloeosporioides* produces specialized infection vesicles formed over or within the stomata, and infection hyphae enter mulberry leaves through the stomata. After penetration, *C. gloeosporioides* can initiate two postinfection strategies, intracellular hemibiotrophy and subcuticular intramural necrotrophy (Perfect et al., 1999; Silva et al., 2017). Subcuticular intramural pathogens include *C. capsica*, *C. circinans*, and *C. musae*. The majority of other *Colletotrichum* species, such as *C. destructivum*, *C. graminicola*, *C. lagenarium*, *C. lindemuthianum*, *C. orbiculare*, *C. sublineolum*, *C. trifolii*, and *C. truncatum*, exhibit intracellular colonization (Perfect et al., 1999). *C. fructicola* and *C. camelliae* might use both intracellular hemibiotrophy and subcuticular intramural necrotrophy infection processes for colonization of tea plant tissues. In intracellular hemibiotrophy, penetrating structures (infection vesicles) are formed that invade the epidermal cells, and the primary hyphae produce enlarged infection vesicles inside the cells of the epidermis and the mesophyll. At this biotrophic phase, the host cells stay alive and plants do not show any symptoms. This phase is followed by the necrotrophic phase, in which secondary hyphae invade the interior of infected cells and neighbouring cells, secreting enzymes that kill them (Münch et al., 2008; Perfect et al., 1999). In subcuticular intramural necrotrophy, the host cuticle is penetrated and fungal hyphae spread subcuticularly within the walls of the host epidermal cells, without penetrating the protoplasm. Subsequently, the hyphae initiate the destruction of the colonized tissues (Perfect et al., 1999) (Figure S1).

### 2.3 The function of *C. gloeosporioides* genes involved in differentiation and pathogenicity

**FIGURE 1:**
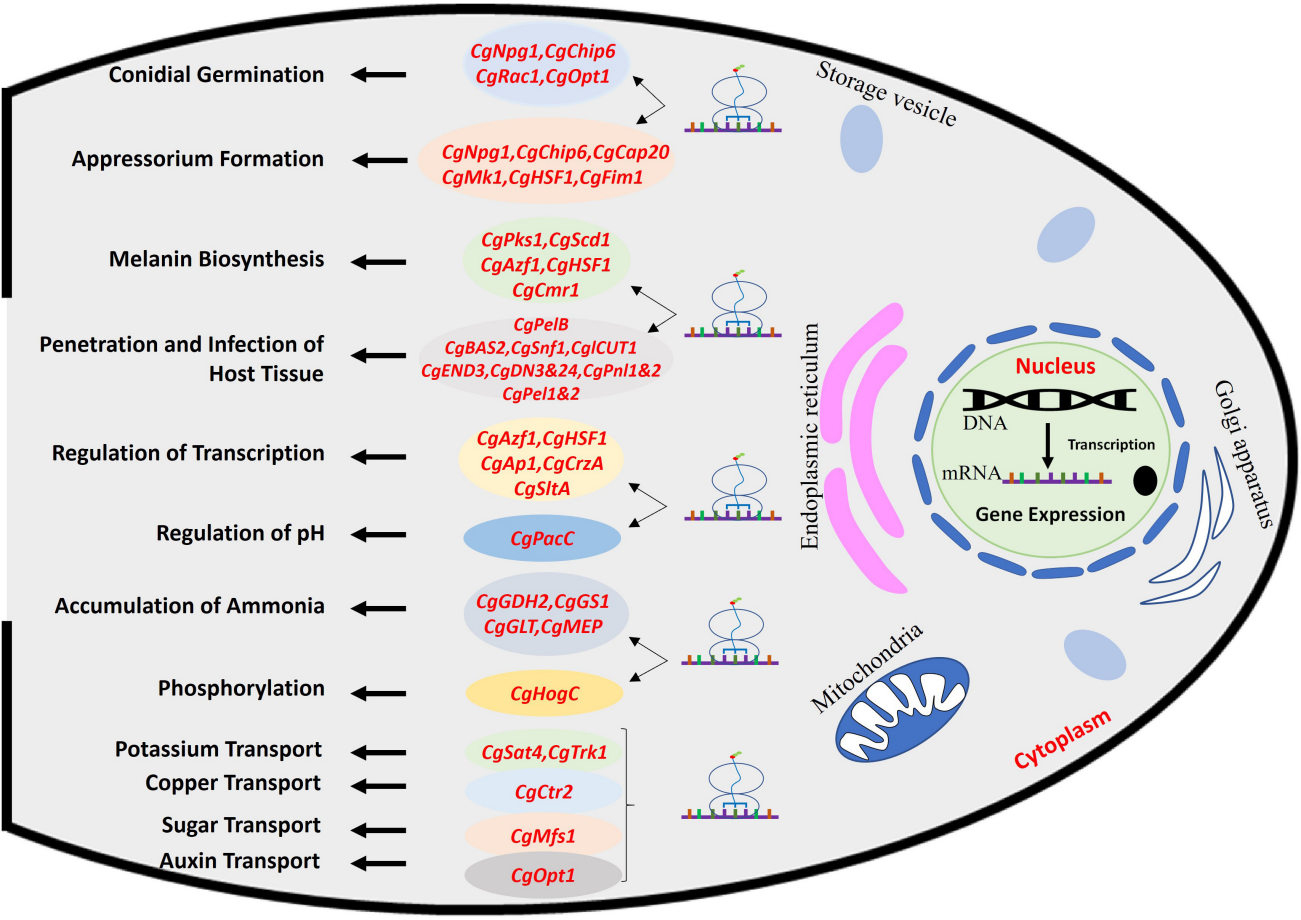
*Colletotrichum gloeosporioides* genes involved in various biological functions that are responsible for pathogen differentiation and pathogenicity at different stages of fungal–plant interaction. The fungal colonization in plant tissue requires expression of several genes in *C. gloeosporioides*. These protein‐encoding genes are involved in the regulation of conidial germination, appressorium formation, melanin biosynthesis, penetration and infection of host, phosphorylation, ammonia accumulation, ion transport, and pH‐controlled genes.

During colonization of host tissue, several genes of *C. gloeosporioides* are involved in various stages of infection processes, which include conidiation, appressorium morphogenesis, melanization and penetration, biotrophy, necrotrophy, and various transport activities (Sharma & Kulshrestha, 2015). The generation and development of conidia requires direct expression of several key regulators for proper vegetative growth, the assembly of the conidiophore, and spore maturation. Recently, Liang et al. (2021) found that the function of *CgNpg1* (novel pathogenic gene 1) in *C. gloeosporioides* is essential for mycelial growth, conidiation, the development of invasive structures such as conidial germination, appressorium formation, and pathogenicity. *CgChip6* encodes a novel fungal virulence factor, sterol glycosyl transferase, that is induced by hard surface contact of the conidia and is involved in conidial germination and appressorium formation (Kim et al., 2002). *CgRac1* is a major regulator of the asymmetric development of *C. gloeosporioides* and encodes Rho GTPase protein, which is abundant in conidia and hyphal tips, and is involved in the regulation of spore germination (Nesher et al., 2011). *CgOpt1*, a putative oligopeptide transporter, is necessary for full virulence and its expression is enhanced in spore germination and reduced again in mycelial development (Chagué et al., 2009). *CgCap20* is one of the genes expressed uniquely in *C. gloeosporioides* during appressorium formation induced by the host signal; it encodes a perilipin homologue protein, which is involved in functional appressoria development (Hwang et al., 1995; Lin et al., 2018). The mitogen‐activated protein kinase (MAPK) *CgMk1* plays a critical role in appressorium formation, melanin biosynthesis, and virulence. *CgMk1* is involved in two developmental processes in the differentiation into appressorium: polarized cell division and differentiation of the germ tube into an appressorium (He et al., 2017; Kim et al., 2000). *CgHSF1* is involved in the pathogenicity of *C. gloeosporioides* through the activation of melanin biosynthesis genes and the regulation of appressorium formation (Gao et al., 2022). Moreover, an actin cross‐linking protein fimbrin homologue (*CgFim1*) is required for the formation of the pore wall overlay, pore contraction, and the extension of a penetration peg, and is also involved in the regulation of the actin cytoskeleton in the hypha and appressorium. *CgFim1*‐mediated F‐actin interacts with septin to regulate appressorium development in *C. gloeosporioides* (Zhang et al., 2022) (Figure 1). These protein‐encoding genes play important roles in conidial germination and appressorium formation during colonization of *C. gloeosporioides* in host tissue.

Melanization of the appressorium is needed by the fungus to penetrate the host tissue, which uses  high pressure for mechanical penetration (Howard & Ferrari, 1989). Previous studies have shown that fungal transcription factors such as heat shock transcription factor (*CgHSF1*) WD40‐repeat protein (*CgCmr1*), polyketide synthase (*CgPks1*), and scytalone dehydratase (*CgScd1*), are involved in the pathogenicity of *C. gloeosporioides* through the activation of melanin biosynthesis genes and the regulation of appressorium formation (Gao et al., 2022; Wang et al., 2020, 2021b). Another transcription factor, C2H2 zinc‐finger protein (*CgAzf1*), regulates the expression of genes involved in the MAPK (*CgMk1*), cyclic adenosine monophosphate‐dependent protein kinase A (cAMP‐PKA), and melanin biosynthesis pathways, and plays an important role in melanin production, conidial development, and infection (Li et al., 2019). A bZIP transcription factor (*CgAP1*), calcineurin‐responsive transcription factor (*CgCrzA*), and cation‐stress‐responsive transcription factors (*CgSltA* and *CgCrzA*) are involved in cell wall integrity, morphogenesis, and pathogenicity in *C. gloeosporioides* (Dubey et al., 2016; Li et al., 2017) (Figure 1). These fungal‐specific transcription factors might orchestrate gene expression control to promote melanization of the appressorium and fungal colonization of the host.

For penetration into the host, the appressorium differentiates from a terminal swelling of a germ tube, and an infection peg develops from the base of the appressorium (Goodwin & Chen, 2002). *CgBAS2* encodes biotrophy‐associated secreted protein, which is involved in the process of the pathogen penetrating into the host tissue and is required for the secretion of a series of proteins for the pathogenicity of *C. gloeosporioides* (An et al., 2018). A sucrose nonfermenting (SNF1)‐related protein kinase, *CgSnf1*, plays a regulatory role in coordinating the alterations in fungal energy status and extracellular enzyme production associated with successful penetration and infection of host tissue (Goodwin & Chen, 2002). *CgEnd3*, an endocytosis‐related protein, plays pleiotropic roles in endocytosis, calcium signalling, cell wall integrity, appressorium formation, penetration, and pathogenicity (Wang et al., 2021a). *CglCUT1* encodes cutinase, an extracellular enzyme that destroys the cuticle of aerial plant parts and is thus important for pathogenicity (Wang, Chen, et al., 2016). *CgDN3* plays an important role at the early stages infection and is required to avert an HR by a compatible host, and *CgDN24* is necessary for normal hyphal development (Stephenson et al., 2005). Three genes, *CgPnl‐1&2*, *CgPel‐B*, and *CgPel1&2*, encoding pectin lyase, pectate lyase, and pectic lyase, respectively, are required for cell wall degradation, penetration, and colonization. The expression of *CgPel‐B*, *CgPnl2*, *CgPel1*, and *CgPel2* significantly increases in the necrotrophic phase of infection (Shih et al., 2000; Wei et al., 2002; Yakoby et al., 2001) (Figure 1). These genes might coordinately regulate the cell wall degradation, penetration, and infection of host tissue.

Host tissue alkalinization via ammonia accumulation is the basic signal for the activation of *CgPacC*, a transcription regulator of pH‐controlled genes that is essential for successful colonization by regulating the expression of transporters, antioxidants, and cell wall‐degrading enzymes (Alkan et al., 2013). In plant pathogens, transporters play an essential role in protection against plant defence mechanisms. One of the major classes of transporter is the major facilitator superfamily (MFS) transporter (*CgMfs1*), which is very similar to hexose transporters required for sugar transport, resistance to oxidative stress, and the pathogenicity of *C. gloeosporioides* (Liu et al., 2021). *C. gloeosporioides* requires copper at the initial stages of pathogenesis and germination, and potassium for maintaining cell shape, membrane potential, intracellular pH, and enzyme activity (Barhoom et al., 2008; Pan et al., 2022). *CgCtr2*, encoding a vacuolar copper transporter, is important in providing copper to copper‐dependent cytosolic activities, thereby regulating cellular copper balance during this process (Barhoom et al., 2008). *CgSat4*, encoding a serine/threonine kinase, is involved in K^+^ uptake by regulating the accurate localization and expression of the potassium transporter *CgTrk1*, and also plays important role in osmotic resistance by altering the phosphorylation level of high osmolarity glycerol response kinase *CgHog1* (Pan et al., 2022). Nitrogen is essential for the biosynthesis of proteins and nucleic acids, which are required for almost all biosynthetic processes, including fungal growth and conidial production. Activation of nitrogen metabolism is a highly complex process. The genes for glutamate dehydrogenase (*CgGDH2*), glutamine synthase (*CgGS1*), glutamate transporter (*CgGLT*), and ammonia permease (*CgMEP*), are involved in the accumulation of ammonia and pathogenicity during colonization by *C. gloeosporioides* (Miyara et al., 2012) (Figure 1). *C. gloeosporioides* requires the activation of nitrogen metabolism for ammonia accumulation and the regulation of pH‐controlled genes for transporter activity, cell wall degradation, penetration, and successful colonization in the host tissue. A list of genes involved in differentiation and pathogenesis of *C. gloeosporioides* is provided in Table S2.

## 3 ANTHRACNOSE DISEASE‐RESISTANCE MECHANISMS IN TEA PLANTS

**FIGURE 2:**
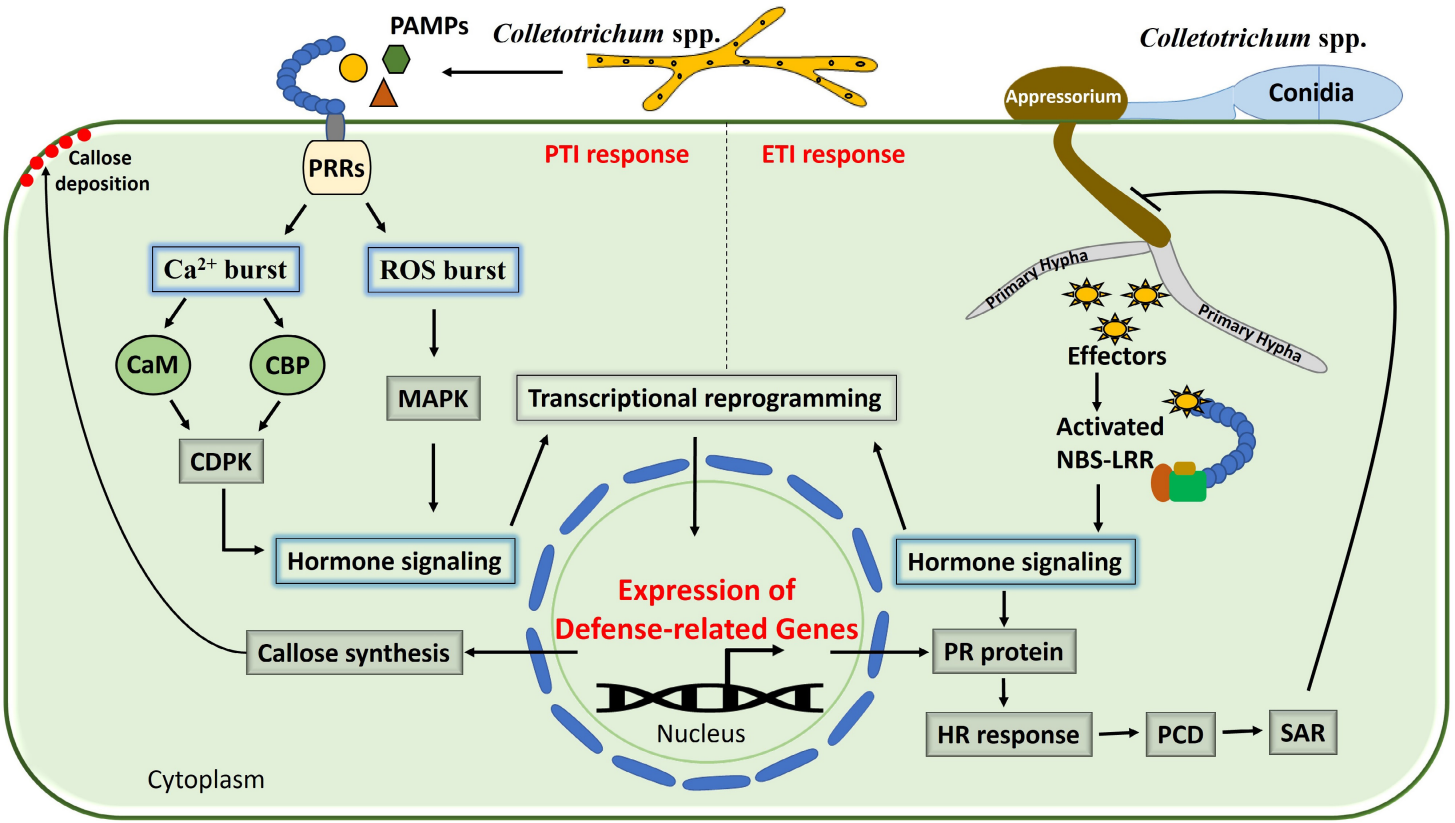
Hypothetical model representing the regulatory mechanisms involved in the resistance of tea plants to anthracnose pathogen. Plants trigger pathogen‐associated molecular pattern (PAMP)‐triggered immunity (PTI) and effector‐triggered immunity (ETI) against pathogens. Recognition of PAMPs by surface pattern recognition receptors (PRRs) activates the PTI defence response, and binding of the NBS‐LRR protein to fungal effector molecules activates the ETI defence response in plants. PTI and ETI extensively share downstream signalling machinery, including MAPKs and Ca^2+^ signalling‐mediated regulation of the reactive oxygen species (ROS) downstream signalling component hydrogen peroxide (H_2_O_2_) for the development of hypersensitive response (HR)‐associated programmed cell death (PCD) to restrict the pathogen. CBP, calmodulin‐binding protein; CDPK, Ca^2+^‐dependent protein kinase; MAPK, mitogen‐activated protein kinase; NBS‐LRR, nucleotide‐binding site leucine‐rich repeat; PR protein, pathogenesis‐related protein; SAR, systemic acquired resistance.

Plants have evolved a sophisticated innate immune system to defend against pathogen infection. Under biotic stresses, plants trigger two layers of immunity against pathogens, namely pathogen‐associated molecular pattern (PAMP)‐triggered immunity (PTI) and effector‐triggered immunity (ETI) (Jones & Dangl, 2006). Once pathogens overcome mechanical barriers to infection, a basal resistance response, PTI, is rapidly activated by extracellular host recognition of PAMPs by surface pattern recognition receptors (PRRs). Effective pathogens have evolved mechanisms to counteract the basal defence by delivering effectors to suppress PTI and cause effector‐triggered susceptibility (ETS) (Dangl et al., 2013; Stotz et al., 2014). In resistant plants, ETI confers a robust resistance response that is initiated by intracellular host recognition of pathogen virulence factors (effectors). To defend against infection, plants employ nucleotide‐binding site, leucine‐rich repeat (NBS‐LRR) proteins that intercept pathogen effectors and stop pathogen growth (Cui et al., 2015). ETI reinstates and amplifies PTI basal transcriptional programmes and antimicrobial defences, which are often associated with an HR and localized cell death, which effectively protects the plant from the threat (Jones & Dangl, 2006). PTI and ETI extensively share downstream signalling machinery mediated by an integrated signalling network (Göhre et al., 2012; Tsuda & Katagiri, 2010) (Figure 2).

### 3.1 PAMP‐triggered immunity

PTI, the first line of active defence in plants, relies on the perception of PAMPs, which represent pathogen‐specific cell wall components. For example, chitin is an essential component of fungal cell walls, and chitin oligosaccharides act as PAMPs in plant cells (Badreddine et al., 2008). Perception of these PAMPs by surface‐localized PRRs activates a plethora of reactions ending in PTI, which contributes to broad‐spectrum resistance (Jones & Dangl, 2006). In plants, PRRs are membrane‐associated receptor‐like kinases (RLKs) and receptor‐like proteins (RLPs). RLKs and RLPs play crucial roles in plant immunity, growth, and development. Plant RLKs are analogous to an animal receptor tyrosine kinase, and are composed of an extracellular ectodomain, a transmembrane domain, and an intracellular kinase domain, whereas RLPs lack the intracellular kinase domain. Leucine‐rich repeat (LRRs), lysin motifs (LysMs), lectin‐like motifs, and epidermal growth factor (EGF)‐like domains are ligand‐binding ectodomains (Saijo et al., 2018). Leucine‐rich repeat receptor kinases (LRR‐RKs) comprise the largest subfamily of transmembrane RLKs in plants that contain LRRs in their extracellular domain. In addition to LRR‐RKs, plant receptor‐like serine threonine kinases (RSTK) play important roles in signalling and plant defence (Goring & Walker, 2004). In tea plants, LRR family proteins are predicted to be up‐regulated in response to *C. camelliae* infection in cultivar LJ43 (Lu et al., 2020), whereas LRR protein kinase, serine/threonine (Ser/Thr) protein kinase, inactive receptor‐like Ser/Thr protein kinase, and LRR receptor‐like Ser/Thr protein kinase are differentially regulated by some microRNAs (miRNAs) in the susceptible cultivars Shuchazao and LJ43 after *C. gloeosporioides* inoculation (Jeyaraj et al., 2019, 2021). However, in resistant cultivar ZC108, serine/threonine‐protein kinase SRK2 is significantly up‐regulated after *C. camelliae* infection (Wang, Hao, et al., 2016), while two cysteine‐rich RLKs (RLK7 and 8), three RLPs (RLP7, 14, and 25), RLK1, receptor Ser/Thr kinase, and LRR protein kinase family protein are specifically activated in response to *C. fructicola* (Wang et al., 2018). Our previous study also identified that LRR receptor‐like protein kinase is down‐regulated, whereas Ser/Thr protein kinase STN7 is up‐regulated in ZC108 (Jeyaraj et al., 2021). The recognition of PAMPs by these PRRs activates the first line of PTI basal defence in tea plants after *Colletotrichum* infection (Figure 2).

Oxidative burst or the generation of reactive oxygen species (ROS) is a typical PTI early cellular response in plants to diverse biotic and abiotic stresses. PAMP‐triggered ROS can regulate programmed cell death (PCD) in plants during pathogen infection and also act as signalling molecules to activate plant defences (Jwa & Hwang, 2017). ROS are produced by various environmental stresses, which are known to activate MAPKs giving a specific response. The MAPK cascade is an evolutionarily conserved signal transduction module involved in transducing extracellular signals to the nucleus for appropriate cellular adjustment (Sinha et al., 2011). The expression of MAPK phosphatase 2 (MKP2), MAPK3, and MAPK5 significantly increased in ZC108 after *C. fructicola* infection (Wang et al., 2018). MKP2 is a regulator of MAPK signalling and PCD for enhanced *Arabidopsis* defence response against biotrophic and necrotrophic pathogens (Vilela et al., 2010). Accordingly, it is possible that MKP2, MAPK3, and MAPK5 might positively activate PCD in tea plant defence against *Colletotrichum* stress (Wang et al., 2018). In addition to oxidative burst, perception of PAMPs by PRRs activates an influx of extracellular calcium ion (Ca^2+^) in the cytosol (Ca^2+^ burst), which is one of the earliest known physiological responses in plants to PAMP perception. After the Ca^2+^ ion affinity of calmodulin, the calmodulin‐binding proteins (CBP) receive signals from the calmodulins to activate Ca^2+^‐dependent protein kinase (CDPK), which could regulate ROS (H_2_O_2_) generation during plant pathogen signalling through phosphorylation of NADPH oxidase (Dodd et al., 2010; Kobayashi et al., 2007). Ca^2+^/CaM also regulates the synthesis of downstream signalling components, such as nitric oxide (NO) and H_2_O_2_, which are essential for the development of the HR (Ma & Berkowitz, 2011). Previous studies in tea plants showed that CBPs and CDPKs are up‐regulated in LJ43 and ZC108, respectively, in response to *C. camelliae* infection (Lu et al., 2020; Wang, Hao, et al., 2016). Callose deposition and various phytohormone signalling systems also play important roles in the tea–*C. camelliae* interaction (Lu et al., 2020). In addition, MAPK1, CDPK‐SK5, and CDPKs are differentially regulated by miRNAs after *C. gloeosporioides* infection in Shuchazao and LJ43 (Jeyaraj et al., 2019, 2021). Plant MAPKs and CDPKs are involved in efficient transmission of specific stimuli, such as hormones, abiotic stresses, and biotic stresses, and also involved in intracellular signalling pathways that play a pivotal role in many essential cellular processes during plant–pathogen interactions (Figure 2).

### 3.2 Effector‐triggered immunity

The main features of the biotrophic phase in *Colletotrichum* species are to suppress the host basal defence response (PTI) for the establishment and maintenance of biotrophy (Perfect et al., 1999). When the first defence system (PTI) is defeated, plants become truly exposed to pathogenic threats. At this stage of infection, *Colletotrichum* species switch from the biotrophic to the necrotrophic phase. The effectors or phytotoxins released from the pathogen initiate a second layer of innate immunity (ETI) in resistant plants (Jones & Dangl, 2006). These pathogen effectors can inhibit PAMP‐PRR‐activated defence signalling networks for successful infection in susceptible plants, leading to ETS (Zipfel, 2014). In the second layer of defence (ETI), resistant plants counteract ETS through the recognition of pathogen effectors by the products of host disease resistance genes that lead to an HR and localized host cell death (Cui et al., 2015). NBS‐LRR proteins are the largest class of resistance proteins that are normally associated with ETI. Binding of NBS‐LRR proteins to fungal effector molecules activates the downstream signalling cascade that leads to the HR in plants. HR is a rapid apoptotic reaction that functions to remove the availability of cytoplasmic nutrients to pathogens in infected cells, and thereby restricts fungal growth and spread (Oren et al., 2016). Previously, it was predicted that the expression of coiled‐coil (CC)‐NBS‐LRR resistance protein, NBS‐LRR resistance protein, NBS‐LRR resistance protein (RPS2), and NBS‐LRR disease‐resistance protein (RPM1) would increase in the leaves of ZC108 after *C. camelliae* inoculation (Wang, Hao, et al., 2016). NB‐ARC domain‐containing disease‐resistance protein and LRR‐NB‐ARC domain‐containing disease‐resistance protein were predicted to be up‐regulated in the resistant cultivar ZC108 in response to *C. fructicola* (Wang et al., 2018). In response to *C. gloeosporioides* infection, TIR‐NBS‐LRRs targeted by miRNAs are differentially expressed between LJ43 and ZC108 (Jeyaraj et al., 2021). Interestingly, Wang et al. (2018) demonstrated that activated R genes regulate MAPK and Ca^2+^ signalling pathways for the continuous production of ROS (H_2_O_2_) in the peroxisome, and then trigger HR‐associated PCD and H_2_O_2_ accumulation around the hyphal infection sites. Activated R genes also regulate the thickening of cell wall tissue to defend against hyphal growth in tea plants. The results of this study suggested that MAPK‐mediated accumulation of ROS in the resistant cultivar ZC108 is associated with the increased expression of peroxin 11a and the inhibition of peroxidase 2, a negative regulator of H_2_O_2_ during *C. fructicola* infection. In addition, wall‐associated kinase 3, a cell signalling receptor, was up‐regulated, and H_2_O_2_ significantly accumulated in the leaves of ZC108 around *C. fructicola* hyphal infection sites, suggesting H_2_O_2_ could regulate cell wall strengthening and activate signalling to resist *C. fructicola* attack (Wang et al., 2018). These resistance proteins could recognize the effectors of *Colletotrichum* species in ZC108, thereby activating the MAPK and Ca^2+^ signalling‐mediated HR‐associated PCD to restrict the pathogen in tea plants (Figure 2).

### 3.3 Phytohormone‐mediated plant defence response against *Colletotrichum* stress

#### 3.3.1 SA signalling

**FIGURE 3:**
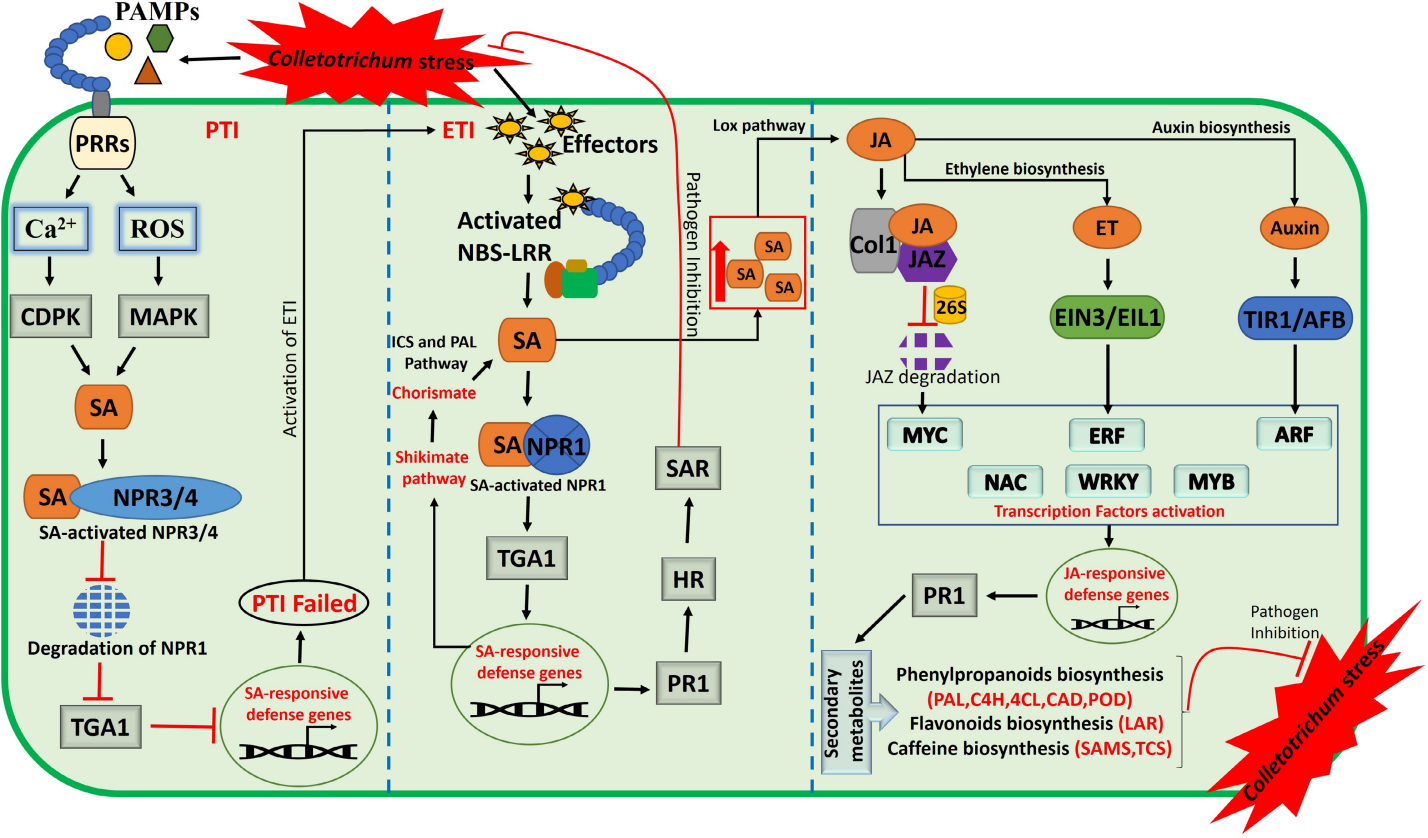
Hypothetical model explaining phytohormone‐mediated molecular mechanisms in the tea plant defence response against anthracnose caused by *Colletotrichum* spp. Phytohormone (salicylic acid [SA], jasmonic acid [JA], ethylene [ET], and indole acetic acid [IAA, auxin]) crosstalk regulates tea plant immune responses to pathogen stress. Negative regulators of SA (NPR3/4) suppress SA gene expression by degradation of NPR1 and interac with TGA1 to suppress pathogen‐associated molecular pattern (PAMP)‐triggered immunity (PTI) defence response to *Colletotrichum*. Failure of PTI and recognition of effectors by NBS‐LRR in resistant plants triggers the biosynthesis of SA, which activates the SA signalling genes NPR1, TGA, and PR1 to regulate tea plant defence against anthracnose infection. The high concentration of SA in effector‐triggered immunity (ETI) might activate the ET/JA signalling and auxin components. The activation of transcription factors (AP2/ERF, TGA1, MYC2, ARF, MYB, WRKY, and NAC) by JA/ET/auxin signalling triggers the expression of a specific set of PR genes and the biosynthesis of secondary metabolites to restrict the pathogen. 26S, 26S proteasome; CDPK, Ca^2+^‐dependent protein kinase; HR, hypersensitive response; ICS, isochorismate synthase; JAZ, jasmonate ZIM‐domain protein; Lox, lipoxygenase; MAPK, mitogen‐activated protein kinase; NBS‐LRR, nucleotide‐binding site leucine‐rich repeat; PAL, phenylalanine ammonia‐lyase; PRR, pattern recognition receptor; ROS, reactive oxygen species; SAR, systemic acquired resistance

Plants produce a combination of phytohormones, such as SA, JA, and ethylene (ET), in response to biotic and abiotic stress. Studies indicate that multiple levels of crosstalk between these phytohormones play a central role in the regulation of plant immune responses to biotic stress (Fujita et al., 2006; Spoel & Dong, 2008). These defence responses are considered to be dependent on the pathogen lifestyle and the genetic constitution of the host. SA is a simple phenolic compound that participates in the regulation of various metabolic pathways in plants. It is considered to play an important role in improving plant stress resistance. SA is synthesized by two enzymatic pathways, isochorismate synthase (ICS) and the phenylalanine ammonia‐lyase (PAL) pathway. The primary metabolite of these pathways is chorismate, the endproduct of the shikimate pathway, to produce SA (Dempsey et al., 2011). During stress conditions, >90% of stimulated SA was synthesized through ICS (Chen et al., 2009). Generally, SA readily undergoes glucosylation, methylation, amino acid conjugation, sulphonation, hydroxylation, and so on to form its derivatives, but most of these are inactive compounds (Dempsey et al., 2011). However, SA also acts as a chemical messenger in regulating various plant biological processes at relatively low concentration. The main storage form of SA in plants is the glucosylated form (SAG), which can potentially be converted back to SA through enzymatic reactions (Dean & Delaney, 2008; George Thompson et al., 2017). A methylated derivative (methyl salicylate, MeSA) is also inactive, but it is volatile and can readily diffuse through membranes. Volatilization of SA through MeSA synthesis can help plants excrete SA outside of the cell in which it is synthesized for eventual diffusion out of the plant (Park et al., 2007; Ross et al., 1999). This mechanism helps plants to maintain SA at a low concentration in cells, whereas a high concentration of SA might damage active plant cells and cause death (Cronjé et al., 2004). When there is no encounter with pathogens or stress, plant cells accumulate a relatively low concentration of SA; this might be due to the expression of nonexpresser of pathogenesis‐related 3/4 genes (*NPR3/4*), which serve as negative regulators of SA and suppress SA gene expression by directly interacting with plant‐specific TGA transcription factors (TGACG motif‐binding factor), members of the bZIP family. This SA‐activated NPR3/4 causes the proteasome degradation of NPR1, a positive regulator of SA, thus it negatively regulates defence (Castelló et al., 2018) (Figure 3).

In tea plants, *Colletotrichum* eventually secrete PAMPs. Recognition of PAMPs by PRRs mediates PTI defence mechanisms, which stimulate several signals, such as generation of ROS, Ca^2+^ influx, transient activation of MAPKs, and the production of SA (Tsuda et al., 2009). Even though there is accumulation of endogenous SA to induce internal defence signals for plant immunity, other siganlling phytohormones, JA and ET, negatively regulate the SA defence role at the transcriptional level (Spoel et al., 2007; Zheng et al., 2012), which further makes tea plants more susceptible to *Colletotrichum*. Failed PTI immune response inevitably makes tea plants more vulnerable to pathogen attack, which initiates ETI to induce localized host cell death during the necrotrophic phase (Jeyaraj et al., 2019; Jones & Dangl, 2006). At this phase, HR prevents further pathogen infection in tea plants by activating systemic acquired resistance (SAR), which might protect tea plants against pathogens. It was previously reported that SAR is dependent on SA signalling and the systemic expression of pathogenesis‐related (PR) genes. Interestingly, Shi et al. (2019) reported that the pathways of Ca^2+^ signalling and SA signalling in KEGG analysis were strongly responsive to anthracnose infection in tea plants. The results of this study showed that in anthracnose‐infected leaves, the genes involved in the SA signalling pathway, such as NPR1, TGA, and PR1, were significantly up‐regulated, and the levels of free SA, bound SA, and total SA also significantly increased, suggesting PR1 and endogenous SA act as a key compounds that play a pivotal role in the activation of tea defence to anthracnose (Shi et al., 2019). This is consistent with previous findings in strawberry in which, after infection with *C. gloeosporioides*, the expression of heat‐shock protein (HSP17.4) activated the up‐regulation of response genes NPR1, TGA, and PR‐1 of the SA signalling pathway and synergistically suppressed downstream signals of JA, contributing to resistance of the plants to infection (Fang et al., 2021). Interestingly, TGA1 from *Populus euphratica* (poplar) enhances disease resistance against *C. gloeosporioides* by directly regulating *PeSARD1* (Systemic Acquired Resistance Deficient 1), which is an important regulator for SA biosynthesis, thus this mechanism elevates SA levels, thereby activating the expression of PR1 (Yang et al., 2022). SAR protective action mainly depends on SA and NPR‐1. NPR1 is the central regulator of the SA signalling pathway and also functions as a co‐activator for SA‐responsive genes. Higher SA levels can induce the monomerization of NPR1 and induce NPR1‐dependent gene expression through direct interactions with TGA transcription factors. This direct binding of NPR1 to SA effectively suppresses the action of NPR3 and NPR4 on SA, which in turn significantly enhances the accumulation of SA‐induced NPR1‐dependent PR genes (Loon & Strien, 1999) (Figure 3). Generally, fungal pathogens produce a blend of hydrolytic enzymes, such as cutinases, pectinases, cellulases, and proteases, that hydrolyse plant cell walls. The main role of the PR genes is to restrict pathogen invasion and replication by producing antifungal proteins, which leads to HR‐induced cell death by activating SAR. SAR is a mechanism of induced defence that confers long‐lasting protection against a broad spectrum of microorganisms, which requires the SA signal molecule (Jeyaraj et al., 2019). Thus, SA plays a crucial role in PTI, ETI, and SAR in tea plants against *Colletotrichum*.

#### 3.3.2 ET/JA signalling

Immune system resistance is dependent on ET/JA signalling and the activation of JA/ET‐related gene expression (Mur et al., 2006). JA/ET signalling is activated to regulate the stress response when plants are infected by necrotrophic fungi and insects (Song et al., 2014). Generally, JA is a derived form of α‐linolenic acid, a component of chloroplast membranes. It is synthesized via the oxylipin biosynthesis pathway through oxidation, and is stored in its endogenous bioactive form JA‐Ile (Ruan et al., 2019). The key enzymes in the JA biosynthesis pathway include LOX2 (lipoxygenase2), AOS (allene oxide synthase), AOC2 (allene oxide cyclase2), and OPR3 (12‐oxo‐phytodienoate reductase3) (Leon‐Reyes et al., 2010). ET is synthesized from methionine. Methionine is converted to ethylene through the intermediates *S*‐adenosylmethionine (SAM) and 1‐aminocyclopropane‐1‐carboxylic acid (ACC). The ET biosynthesis pathway consists of two dedicated steps: SAM is converted into ACC by ACC‐synthase (ACS), and ACC is converted into ethylene by ACC‐oxidase (ACO) (Houben & Van de Poel, 2019). In tea plants, Lu et al. (2020) found that several genes associated with ET, JA, and IAA biosynthesis are significantly up‐regulated during the late interaction between tea plants and *C. camelliae*. The precursors of JA, polyunsaturated fatty acids such as α‐linolenic acid and linoleic acid, are activated into hydroperoxides by LOX. Subsequently, hydroperoxides are catalysed by various enzymes and used in many pathways. The intermediate metabolites 13(S)‐HPOT and 12‐oxo‐phytodienoic acid (12‐OPDA) significantly increase to generate JA (Lu et al., 2020). The key genes SAM‐1, ACS, and ACO involved in ET biosynthesis and the JA biosynthesis‐related genes AOS and AOC were up‐regulated at 72 h postinfection (Lu et al., 2020). The JA/ET signal is perceived by receptor proteins and transduced through a phosphorylation cascade that leads to transcription factor activation and consequently JA/ET‐related gene expression (Ku et al., 2018). JA and ET interaction activates COI1‐dependent degradation of jasmonate ZIM‐domain (JAZ) proteins and enhances the transcriptional activity of ET‐stabilized transcription factors (EIN3/EIL1) (Zhu et al., 2011). Previous studies have reported that MYC2 is a core transcription factor that positively regulates the JA signalling pathway (Chen et al., 2012; Kazan & Manners, 2013), and transcription factor AP2/ERF has important regulatory functions in plant defences against pathogens and acts as a key regulatory hub in ET signalling and stress response (Müller & Munné‐Bosch, 2015). Interestingly, the major downstream regulatory factors of ET/JA signalling pathways, such as EIN3, ethylene response factors (ERF1), and basic‐helix–loop–helix (bHLH) transcription factor (MYC2), are significantly up‐regulated during the interaction of tea plants with *C. camelliae* (Lu et al., 2020). In response to *C. fructicola*, the expression of the ET‐responsive element binding protein‐coding gene (ERF) is specifically increased in resistant tea plants (Wang et al., 2018). Wang, Hao, et al. (2016) observed that the JAZ proteins and APETALA2/ERFs (AP2/ERFs) were differentially expressed in tea plants after *C. camelliae* inoculation (Figure 3). These differentially expressed genes (EIN3, ERF, and MYC2) related to ET/JA biosynthesis and signalling may play important roles in the defence response of tea plants to *Colletotrichum*.

#### 3.3.3 Auxin signalling

Auxins are plant hormones that play a critical role in plant responses to environmental stresses. During plant defence, the auxin and SA pathways act in a mutually antagonistic manner, whereas auxin and JA signalling share many commonalities (Koornneef & Pieterse, 2008). The mutual antagonism between the signalling of auxin and SA impacts on plant growth and defence. The high level of SA significantly reduces the reservoir of active IAA, and thus induces the defence mechanism instead of growth. In normal conditions, auxin‐mediated suppression of SA responses will happen, which can enhance growth for better yield (Naseem et al., 2015). Furthermore, there is a synergistic relationship between auxin and JA. During pathogen infection, pathogens generate auxin signals that are perceived by auxin receptors (TIR1/AFB) and transmitted through signalling pathways to activate the auxin response factor (ARF) gene. The JA signal was shown to increase auxin biosynthesis by inducing anthranilate synthase (ASA1) and YUCCA (YUC8 and YUC9) genes in plants (Hentrich et al., 2013). Interestingly, Lu et al. (2020) found that the intermediate products involved in polyunsaturated fatty acid metabolism and IAA increased during the late stages of the tea–*Colletotrichum* interaction. This study also reported that the accumulation of JA and IAA significantly increased in diseased leaves at 72 h postinoculation. In addition, a previous study has shown that genes encoding auxin resistant 2, auxin‐responsive protein, and SAUR‐like auxin‐responsive protein are induced by *C. fructicola*, and the expression levels of the auxin efflux carrier family gene and the SAUR‐like auxin‐responsive protein family gene are specifically increased in resistant tea plants (Wang et al., 2018). In the response of tea plants to *C. camelliae*, the transcription factor ARF9 was significantly down‐regulated, while the four transcripts encoding indole acetaldoxime dehydratase, involved in IAA synthesis, were differentially regulated (Lu et al., 2020). Wang, Hao, et al. (2016) found that the auxin signal transduction‐related gene auxin response factor (ARF), SAUR family protein, and auxin influx carrier (AUX1) showed significant differences in transcript abundance in ZC108 compared to LJ43 independent of pathogen infection conditions. In response to *C. gloeosporioides*, the expression level of csn‐miR160c target transcript ARF5 significantly increased in infected leaves at 10–13 days postinoculation, and auxin signalling F‐box proteins AFB2 and ARF17/18 increased in the resistant cultivar ZC108 (Jeyaraj et al., 2019, 2021) (Figure 3). The activated ARF positively regulates phytoalexin biosynthesis, which results in resistance to pathogens. The enhancement of JA‐dependent defence signalling may be part of the auxin‐mediated defence mechanism that causes activation of phytoalexin to generate plant resistance to *Colletotrichum*.

### 3.4 Transcription factors involved in plant defence against *Colletotrichum* infection

Transcription factors are proteins that are involved in plant defence regulation through acting as mediators by perceiving stress signals and directing the expression of stress‐responsive genes. The activation/inactivation of transcription factors triggers diverse metabolic pathways, allowing plants to produce defensive proteins, metabolites, plant hormones, and/or transcriptional and posttranscriptional modifications (Chacón‐Cerdas et al., 2020). In response to *Colletotrichum* infection, seven different plant‐specific transcription factors, AP2/ERF, TGACG motif‐binding factor (bZIP), MYC2 (bHLH), ARF, MYB, WRKY, and NAC, have been reported to be significantly involved in disease resistance by regulating defence‐associated genes. Among these, the crucial roles of AP2/ERF, TGA, MYC2, and ARF in regulating *Colletotrichum* stress response in tea plants has been covered above in detail. WRKY and MYB are up‐regulated in tea plants on *C. camelliae* infection (Lu et al., 2020; Wang, Hao, et al., 2016), while GRAS family transcription factor and NAC domain containing protein 4 are specifically activated in resistant tea cultivars in response to *C. fructicola* (Wang et al., 2018). NAC and WRKY transcription factors belong to a large family of plant‐specific transcription factors in higher plants. A previous study by Shan et al. (2016) showed that the banana fruit NAC transcription factor MaNAC5 cooperates with MaWRKY1 and MaWRKY2 to regulate the expression of a specific set of PR genes that are involved in SA‐ and methyl jasmonate (MeJA)‐induced resistance against *C. musae*. MYB transcription factors function as activators of structural genes involved in the biosynthesis of antifungal phenylpropanoid compounds, the largest class of natural products, including flavonoids, anthocyanins, monolignols, and tannins, which are indispensable to several developmental pathways and stress responses (Pratyusha & Sarada, 2022). For example, Ibraheem et al. (2015) found that a sorghum MYB transcription factor, involved in the biosynthesis of 3‐deoxyanthocyanidins, enhances resistance against anthracnose caused by *C. graminicola* in maize. Interestingly, blocking miR858 activity by target mimics results in up‐regulation of the flavonoid‐specific target genes *AtMYB11*, *AtMYB12*, and *AtMYB111*, as well as genes upstream of the flavonoid branch in the phenylpropanoid pathway (e.g., *P* *henylalanine ammonia‐lyase* [*PAL4*], *Cinnamate‐4‐hydroxylase* [*C4H*], and *4‐Coumarate‐CoA‐ligase* [*4CL*]) (Camargo‐Ramírez et al., 2018) (Figure 3). Molecular mechanisms of transcription factor‐mediated plant defence regulation against *Colletotrichum* are still unclear in tea plants, therefore further studies are needed to investigate the significant function of these transcriptional regulators involved in *Colletotrichum* stress signalling and downstream defence gene expression.

### 3.5 Secondary metabolites play crucial roles in tea plants against *Colletotrichum* infection

In plant defence systems, phenylpropanoids are a diverse group of polyphenolic compounds, including flavonoids, lignans, tannins, phenolic acids, and coumarins, which are widely distributed secondary metabolites that possess direct and indirect antimicrobial activities, and are involved in plant development and the disease response. During interaction with pathogenic organisms, plant defence mechanisms facilitate the biosynthesis and activation of these compounds through PAMP recognition by PRRs (Ahuja et al., 2012). It is well known that tea plant leaves are rich in polyphenols and caffeine. Catechins, which include epigallocatechin‐3‐gallate (EGCG), epicatechin (EC), epicatechin‐3‐gallate (ECG), epigallocatechin (EGC), catechin (C), and gallocatechin (GC), are the dominant flavonoids of tea plants (Musiał et al., 2020; Wang, Hao, et al., 2016). Flavonoid biosynthesis and phenylalanine metabolism play important roles in tea plant defence against anthracnose (Wang et al., 2018; Wang, Hao, et al., 2016). Wang, Hao, et al. (2016) investigated changes in total phenol, catechins, and caffeine in the leaves of LJ43 and ZC108 after *C. fructicola* infection and found that the total phenolic content was increased by *C. fructicola*. In addition, the content of (+)‐C, (−)‐EGC, (−)‐EGCG and caffeine content was markedly increased in young tissue of resistant cultivar ZC108, suggesting that these compounds play important roles in the resistance of tea plants to anthracnose. This study also showed that the expression levels of a flavonoid biosynthesis‐related gene (leucoanthocyanidin reductase, *LAR*) and caffeine biosynthesis‐related genes (*S*‐adenosylmethionine synthetase, *SAMS*; tea caffeine synthase, *TCS1*) were induced in the young ZC108 tissues inoculated with *C. fructicola* (Wang, Hao, et al., 2016). The leaf tea polyphenol content increased in both ZC108 and LJ43 after inoculation with *C. camelliae*, but the expression of genes involved in phenylpropanoid biosynthesis, such as cinnamyl alcohol dehydrogenase (*CAD*), peroxidase (*POD*), β‐glucosidase (*GUS*), coniferyl‐aldehyde dehydrogenase (*ALDH*), and phenylalanine ammonia‐lyase (*PAL*), was higher in ZC108 than in LJ43, suggesting that phenylpropanoid biosynthesis pathway‐related gene expression might underlie ZC108 resistance to *C. camelliae* (Wang, Hao, et al., 2016) (Figure 3). In *Stylosanthes* the flavonoid biosynthetic genes and accumulation of flavonoid compounds was significantly induced during *C. gloeosporioides* infection. The induced flavonoids, such as phloretin, naringenin, apigenin, daidzein, quercetin, and kaempferol, showed an inhibitory effect on the in vitro growth of *Colletotrichum* strains through the suppression of the mycelial growth and conidial germination (Jiang et al., 2021). Following the perception of *C. sublineolum* invasion, sorghum (*Sorghum bicolor*) launches a complex arsenal of chemical defences. De novo biosynthesis of the antifungal 3‐deoxyanthocynidin phytoalexins, apigeninidin, luteolinidin, and related conjugates were found to differentially accumulate; these metabolites have been shown to exhibit biological activity against fungal pathogens and significant inhibition of spore germination (Cheynier et al., 2013; Tugizimana et al., 2019). These previous studies have indicated that caffeine and polyphenolic compounds are potentially involved in chemical defence mechanisms in tea plants against *Colletotrichum* stress.

**FIGURE 4:**
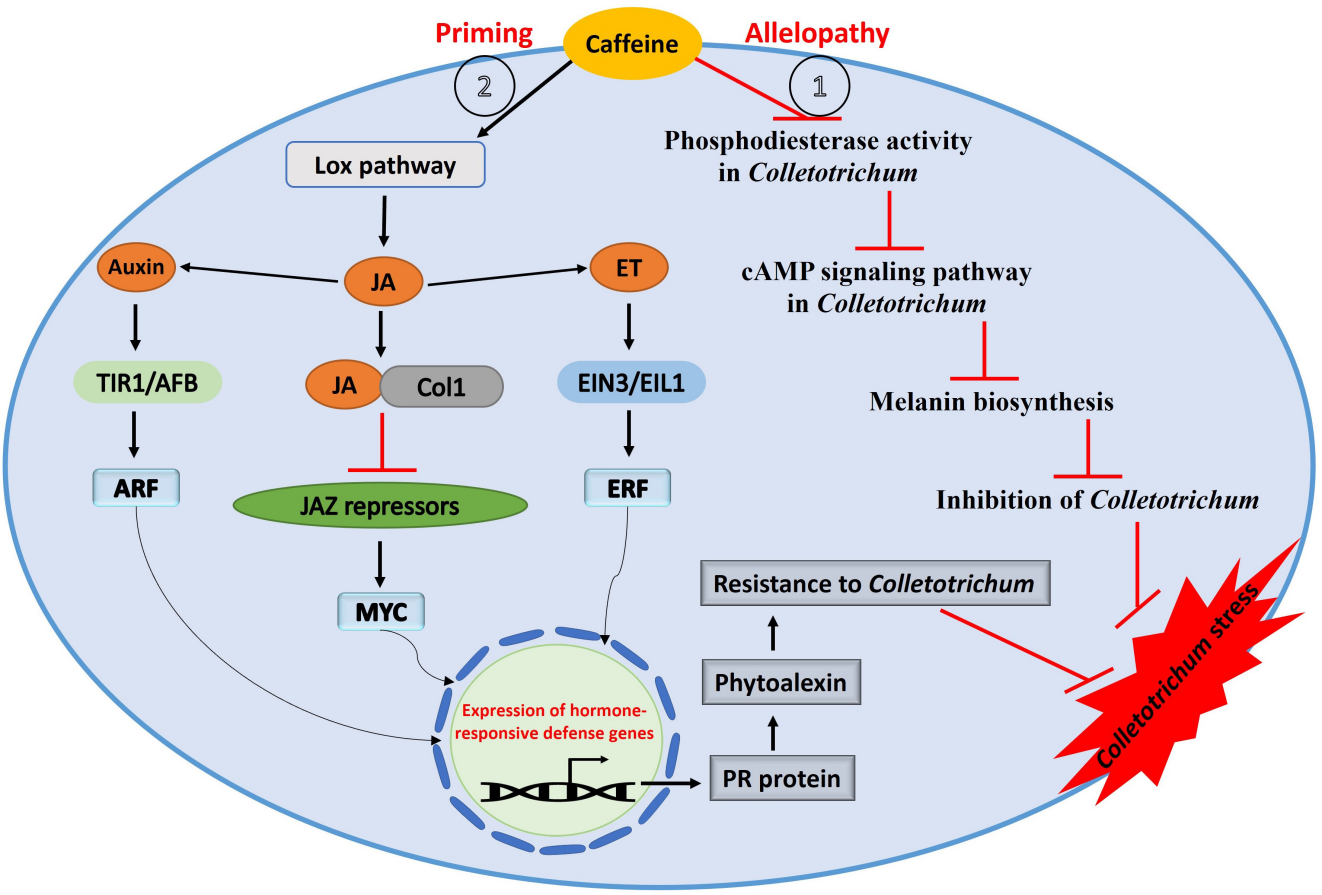
A schematic model showing the exogenous caffeine‐induced defence response involved in the determination of tea plant resistance to *Colletotrichum* spp. Exogenous caffeine directly inhibits the pathogen by its toxicity (allelopathy) through the regulation of phosphodiesterase activity, the cAMP signalling pathway, and melanin biosynthesis in *Colletotrichum*. Caffeine indirectly stimulates the plant defence response against the pathogen through the regulation of the jasmonic acid (JA)/ethylene (ET) signalling pathway and auxin signal components (priming).

Caffeine, an important purine alkaloid naturally found in fresh tea leaves, is synthesized from purine metabolites as a secondary metabolite. Tea caffeine synthase is an enzyme that possesses the 1‐N methyltransferase activity responsible for converting theobromine to caffeine, and it is considered to be the most critical enzyme in the caffeine biosynthetic pathway of tea plants (Kato et al., 2000). Several studies have reported that caffeine can directly restrict the development and growth of pathogenic microbes by its toxicity (allelopathy), and indirectly stimulates the plant defence response by affecting or altering signalling pathways (priming) (Sugiyama et al., 2016). For example, Tsubouchi et al. (1985) reported that exogeneous caffeine directly inhibits the growth of *Aspergillus ochraceus* and its production of the toxic compound ochratoxin A. In *Colletotrichum*, the cAMP signalling pathway was shown to be critical for the formation of melanin, which is essential for an appressorium to penetrate plant cells (Langfelder et al., 2003). Caffeine and its derivative methylxanthine strongly inhibit phosphodiesterase activity, leading to acceleration of cAMP signalling pathways (Weinberg & Bealer, 2001). In tea plants there have only been a few studies focused on the function of caffeine in the tea plant defence mechanism against *Colletotrichum* stress. Wang, Hao, et al. (2016) found that caffeine was induced in *C. fructicola*‐resistant tissues, suggesting that caffeine and genes related to the caffeine pathway may be involved in resistance of tea plants to anthracnose. This study also showed that caffeine strongly inhibited the mycelial growth of *C. fructicola* by affecting mycelial cell walls and plasma membranes. A study by Li et al. (2016) showed that elevated CO_2_ reduced the endogenous caffeine levels in the leaves of tea plants, which increased tea plant susceptibility to *C. gloeosporioides*. This study also suggested that exogenous application of caffeine onto leaves of susceptible tea cultivars induced a defence response against *C. gloeosporioides* infection under elevated CO_2_ through the activation of the lipoxygenase (LOX) pathway and biosynthesis of JA (Figure 4). Our own study confirmed that the effect of exogenous caffeine on tea plants infected with *C. gloeosporioides* is highly dependent on the JA/ET signalling pathway and auxin signal components. It was shown that exogenous caffeine‐induced miRNA‐mediated target genes were actively involved in the regulation of the JA‐mediated pathway, altered the susceptibility of LJ43 against *C.gloeosporioides*, and also enhanced the resistance of ZC108 (Jeyaraj et al., 2021) (Figure 4). Therefore, the crosstalk between these hormone signals and their gene regulatory networks is considered as an important hub in the tea plant responce to pathogen infection.

SAR is dependent on SA signalling and systemic expression of PR genes. However, when exogenous compounds or fungicides are used on infected tea plants, this probably induces defence through the JA/ET signalling pathway against hemibiotrophic pathogens. Increased concentrations of SA certainly have antagonistic effects on tea leaves that trigger cell death and the production of apoplastic ROS (Mur et al., 2006). Increased ROS production probably triggers JA and its derivatives, which are essential to complete the development of SAR (Truman et al., 2007). It is noteworthy that SA and AOS, respectively, share a chloroplastic site for synthesis and activity. AOS is the key enzymatic phase in the biosynthesis of JA. SA is thought to regulate the activity of AOS and thereby activate the JA pathway. When SA levels are high, NPR3 interacts with its substrate NPR1, which deactivates the defence gene expression during ETI, and subsequently stimulates the activation of the JA signalling pathway. Both NPR3 and NPR4 can also interact with JAZ proteins in an SA‐enhanced manner leading to the degradation of JAZs. This results in the activation of de novo JA biosynthesis and amplification of JA signalling through the canonical pathway. Thus, SA could antagonize JA signalling by regulating its key enzyme and also by degrading JA‐responsive transcriptional regulators.

## 4 CONCLUSION AND FUTURE PERSPECTIVES

In recent decades tremendous progress has been made in understanding tea–*Colletotrichum* interactions, driven by new molecular techniques and greater computational power. In this review, we elaborate plant defence systems, including host resistance via physical barriers, PTI, and ETI in tea–*Colletotrichum* interactions. These immune systems enable the perception of pathogen attack as the infection process starts, being the expression of resistance and susceptibility conditioned by the magnitude and/or timing of defence responses. The attempted infection by a pathogen can activate plant immune responses, which include complex histological, cellular, biochemical, and molecular events so that pathogen proliferation or disease spread is limited. Perception of pathogens by plants leads to activation of specific resistance receptors, which eventually stop pathogen proliferation by inducing different immune responses, including Ca^2+^ signalling, nitric oxide and ROS production, alteration in membrane trafficking, transcriptional reprogramming of defence genes, and PCD (HR). The current understanding of the role of phytohormones leading to HR in plant–pathogen interactions hypothesizes that (1) biotrophy is controlled by SA while necrotrophy/hemibiotrophy is controlled by JA/ET, and (2) SA is an antagonist of JA /ET. Anthracnose disease caused by *Colletotrichum* spp. is a hemibiotrophic pathogen. Indeed, tea plants trigger SA signalling during the resistance response against *Colletotrichum* spp. SA signalling further activates the various transcription factors, which trigger defence signals against *Colletotrichum* spp.

Enrichment of tea plant tolerance to anthracnose is essential for high‐quality, safe food. Tea research is a promising research area due to its potential health benefits for humans. Progress in this area is also aided by the degree to which we have identified the molecular mechanisms of stress resistance in tea plants. Crop losses due to anthracnose is a key stimulus for researchers to develop resistant, high‐yielding, high quality cultivars. Although chemical control agents like fungicides and pesticides can be used to fight pathogens, they can also pose severe environmental hazards. Thus, ecofriendly biocontrol strategies are now becoming widely accepted and promising alternatives. Various advanced techniques in bioinformatics and molecular biology provide other approaches for crop improvement and plant defence management. RNA interference (RNAi) or posttranscriptional gene silencing is a mechanism of gene regulation and natural defence responses against various biotic stresses. RNAi technology is an important and exciting field of research in plants for the production of resistant cultivars in advanced agricultural production, and it can also help in the study of complex plant−pathogen molecular interactions RNAi is mainly mediated by different kinds of small RNAs, the most widely studied of which are miRNAs and siRNAs. Two major methods of using RNAi in crop improvement against pathogens are transgene‐mediated host‐induced gene silencing (HIGS) and exogenous double stranded RNA (dsRNA) spray‐induced gene silencing (SIGS). This technology can precisely silence a target gene via a double‐stranded (ds) RNA molecule sharing sequence complementarity with its mRNA (Fire et al., 1998; Wilson & Doudna, 2013), which could be involved in developing resistance against pathogens (Zotti et al., 2018). In SIGS, dsRNA can either be ingested by the fungus from the surface of the plant or it can be taken in by the plant, processed into siRNA, and transferred to the fungus via cross‐kingdom RNA exchange (Song et al., 2018; Wang & Dean, 2020). However, the anthracnose‐causing fungus is not able to absorb dsRNA sprayed on the surface of plant tissues. Thus, sprayed dsRNA cannot be efficiently used against *Colletotrichum* in tea plants. HIGS uses inhibitory dsRNA in the host plant, which confers protection from infection by targeted gene silencing (Koch & Kogel, 2014; Nowara et al., 2010; Price & Gatehouse, 2008). Extensive genomic studies on miRNAs and siRNAs that are involved in the regulation of plant growth and development, signal transduction, protein degradation and response to biotic stresses make it an attractive field of gene silencing research. However, the mechanisms underlying the transfer and uptake of transgene‐derived artificial sRNAs or exogenously applied dsRNA are not fully understood (Werner et al., 2020). Other approaches are overexpressing or suppressing one or more specific miRNAs, depending on whether the target gene(s) has a desirable or an undesirable effect in the host. Overexpression of a miRNA is done by using endogenous processing machinery (DCL1) to process pri‐ and/or pre‐form of miRNA into a mature form, which has its effect on its target gene. In contrast, anti‐miR (short tandem target mimic [STTM]) can be used for the suppression and/or activation of target mRNA. The artificial target mimicry technology, STTM, is used to block or suppress the functions of specific endogenous mature miRNAs in plants (Wang et al., 2019). Thus, this miRNA‐based technology might be used to overexpress or suppress miRNA, or anti‐miRNAs or miRNA mimics in the host plant to target the specific genes involved in defence and immune response and thus might potentially act as a useful technology for crop improvement and protection.

The current research trend is to try to regulate natural plant immune systems to slow down pathogen spread by using new advanced methods of genetic engineering to boost disease resistance in plants. In conventional transgenic methods, genes that encode desired agronomic traits are inserted into the genome at random locations through plant transformation (Lorence & Verpoorte, 2004). These methods typically result in varieties containing foreign DNA. In contrast, genome editing allows changes to the endogenous plant DNA, such as deletions, insertions, and replacements of DNA of various lengths at designated targets (Barrangou & Doudna, 2016). However, rapid identification of novel immune receptor genes, such as through RNA‐Seq and directed molecular evolution, has expanded the pool of deployable genes for enhanced resistance to microbes substantially. Targeted gene insertion through genome editing is expected to play a major role in generating broad‐spectrum resistance against pathogens in the near future. Continuing to alter the receptors necessary to initiate defence responses is probably the best route for developing resistance. NLRs may become a major tool of biotechnology, used to engineer resistance to any pathogen through the CRISPR/Cas9 system. CRISPR‐Cas is an accurate and versatile genome editing tool. In future, CRISPR/Cas9 technology will be used to accurately edit the genome, targeting genes involved in SA signalling, transcription factors, and defence‐related genes, to improve the tea plant immune system and obtain long‐lasting resistance against anthracnose.

In this review, we summarized the *C. gloeosporioides* infection stages and also elaborated the role of various fungal genes involved in its development and pathogenicity in tea plants. Modification of tea plant immunity after *Colletotrichum* infection is illustrated by summarizing changes in SA, JA/ET, and auxin signalling pathways. These phytohormones activate transcription factors like WRKY and MYB, which in turn activate PR genes and secondary metabolites to enhance the plant immunity against the pathogen infection. Finally, we conclude that knowledge about molecular signalling mechanisms involved in the *Colletotrichum–*tea plant interaction offers new insights to develop genetically engineered resistant tea plant cultivars against this pathogen, thereby leading to improved tea plant vitality.

## CONFLICT OF INTEREST STATEMENT

The authors declare they have no conflict of interest.

## Supporting information


**FIGURE S1** Disease cycle of tea plant anthracnose caused by *Colletotrichum* spp. with microscope images displaying different stages of infection caused by the *C. gloeosporioides* on tea leaf. The conidia from necrotic lesions of infected leaves (acervuli) are dispersed by rain splashes or wind. After that, *C. gloeosporioides* can initiate different stages of infection, including conidium attachment on the surface of leaves, germination of conidium, formation of appressorium, and two postinfection strategies (intracellular hemibiotrophy and subcuticular intramural necrotrophy) for successful colonization of plant tissues.

**TABLE S1** The morphological characterization of identified *Colletotrichum* spp. in *Camellia sinensis*

**TABLE S2** List of genes involved in differentiation and pathogenesis of *Colletotrichum gloeosporioides*

## Data Availability

Data sharing is not applicable to this article as no new data were created.

## References

[mpp13354-bib-0001] Ahuja, I. , Kissen, R. & Bones, A.M. (2012) Phytoalexins in defense against pathogens. Trends in Plant Science, 17, 73–90. 22209038 10.1016/j.tplants.2011.11.002

[mpp13354-bib-0002] Alkan, N. , Meng, X. , Friedlander, G. , Reuveni, E. , Sukno, S.A. , Sherman, A. et al. (2013) Global aspects of pacC regulation of pathogenicity genes in *Colletotrichum gloeosporioides* as revealed by transcriptome analysis. Molecular Plant‐Microbe Interactions, 26, 1345–1358. 23902260 10.1094/MPMI-03-13-0080-R

[mpp13354-bib-0003] An, B. , Wang, W. , Guo, Y. , Wang, Q. , Luo, H. & He, C. (2018) BAS2 is required for conidiation and pathogenicity of *Colletotrichum gloeosporioides* from *Hevea brasiliensis* . International Journal of Molecular Sciences, 19, 1860. 29941774 10.3390/ijms19071860 PMC6073657

[mpp13354-bib-0004] Badreddine, I. , Lafitte, C. , Heux, L. , Skandalis, N. , Spanou, Z. , Martinez, Y. et al. (2008) Cell wall chitosaccharides are essential components and exposed patterns of the phytopathogenic oomycete *Aphanomyces euteiches* . Eukaryotic Cell, 7, 1980–1993. 18806214 10.1128/EC.00091-08 PMC2583540

[mpp13354-bib-0005] Barhoom, S. , Kupiec, M. , Zhao, X. , Xu, J. & Sharon, A. (2008) Functional characterization of CgCTR2, a putative vacuole copper transporter that is involved in germination and pathogenicity in *Colletotrichum gloeosporioides* . Eukaryotic Cell, 7, 1098–1108. 18456860 10.1128/EC.00109-07 PMC2446676

[mpp13354-bib-0006] Barrangou, R. & Doudna, J.A. (2016) Applications of CRISPR technologies in research and beyond. Nature Biotechnology, 34, 933–941. 10.1038/nbt.3659 27606440

[mpp13354-bib-0007] Cai, L. , Hyde, K.D. , Taylor, P. , Weir, B.S. , Waller, J.M. , Abang, M. et al. (2009) A polyphasic approach for studying *Colletotrichum* . Fungal Diversity, 39, 183–204.

[mpp13354-bib-0008] Camargo‐Ramírez, R. , Val‐Torregrosa, B. & San Segundo, B. (2018) MiR858‐mediated regulation of flavonoid‐specific MYB transcription factor genes controls resistance to pathogen infection in *Arabidopsis* . Plant and Cell Physiology, 59, 190–204. 29149328 10.1093/pcp/pcx175

[mpp13354-bib-0009] Castelló, M.J. , Medina‐Puche, L. , Lamilla, J. & Tornero, P. (2018) NPR1 paralogs of *Arabidopsis* and their role in salicylic acid perception. PLoS One, 13, e0209835. 30592744 10.1371/journal.pone.0209835 PMC6310259

[mpp13354-bib-0010] Chacón‐Cerdas, R. , Barboza‐Barquero, L. , Albertazzi, F. & Rivera‐Méndez, W. (2020) Transcription factors controlling biotic stress response in potato plants. Physiological and Molecular Plant Pathology, 112, 101527.

[mpp13354-bib-0011] Chagué, V. , Maor, R. & Sharon, A. (2009) CgOpt1, a putative oligopeptide transporter from *Colletotrichum gloeosporioides* that is involved in responses to auxin and pathogenicity. BMC Microbiology, 9, 173. 19698103 10.1186/1471-2180-9-173 PMC2769210

[mpp13354-bib-0012] Chen, R.F. , Jiang, H. , Li, L. , Zhai, Q. , Qi, L. , Zhou, W. et al. (2012) The *Arabidopsis* mediator subunit MED25 differentially regulates jasmonate and abscisic acid signaling through interacting with the MYC2 and ABI5 transcription factors. The Plant Cell, 24, 2898–2916. 22822206 10.1105/tpc.112.098277 PMC3426122

[mpp13354-bib-0013] Chen, Y. , Qiao, W.L. , Zeng, L. , Shen, D. , Liu, Z.L. , Wang, X. et al. (2017) Characterization, pathogenicity, and phylogenetic analyses of *Colletotrichum* species associated with brown blight disease on *Camellia sinensis* in China. Plant Disease, 101, 1022–1028. 30682936 10.1094/PDIS-12-16-1824-RE

[mpp13354-bib-0014] Chen, Z. , Zheng, Z. , Huang, J. , Lai, Z. & Fan, B. (2009) Biosynthesis of salicylic acid in plants. Plant Signaling & Behavior, 4, 493–496. 19816125 10.4161/psb.4.6.8392 PMC2688294

[mpp13354-bib-0015] Cheynier, V. , Comte, G. , Davies, K.M. , Lattanzio, V. & Martens, S. (2013) Plant phenolics: recent advances on their biosynthesis, genetics, and ecophysiology. Plant Physiology and Biochemistry, 72, 1–20. 23774057 10.1016/j.plaphy.2013.05.009

[mpp13354-bib-0016] Cronjé, M.J. , Weir, I.E. & Bornman, L. (2004) Salicylic acid‐mediated potentiation of Hsp70 induction correlates with reduced apoptosis in tobacco protoplasts. Cytometry Part A, 61A, 76–87. 10.1002/cyto.a.20036 15351992

[mpp13354-bib-0017] Cui, H. , Tsuda, K. & Parker, J.E. (2015) Effector‐triggered immunity: from pathogen perception to robust defense. Annual Review of Plant Biology, 66, 487–511. 10.1146/annurev-arplant-050213-040012 25494461

[mpp13354-bib-0018] Dangl, J.L. , Horvath, D.M. & Staskawicz, B.J. (2013) Pivoting the plant immune system from dissection to deployment. Science, 341, 746–751. 23950531 10.1126/science.1236011 PMC3869199

[mpp13354-bib-0019] Dean, J.V. & Delaney, S.P. (2008) Metabolism of salicylic acid in wild‐type, *ugt74f1* and *ugt74f2* glucosyltransferase mutants of *Arabidopsis thaliana* . Physiologia Plantarum, 132, 417–425. 18248508 10.1111/j.1399-3054.2007.01041.x

[mpp13354-bib-0020] Dempsey, D.A. , Vlot, A.C. , Wildermuth, M.C. & Klessig, D.F. (2011) Salicylic acid biosynthesis and metabolism. The Arabidopsis Book, 9, e0156. 22303280 10.1199/tab.0156 PMC3268552

[mpp13354-bib-0021] Dobón, A. , Canet, J.V. , García‐Andrade, J. , Angulo, C. , Neumetzler, L. , Persson, S. et al. (2015) Novel disease susceptibility factors for fungal necrotrophic pathogens in *Arabidopsis* . PLoS Pathogens, 11, e1004800. 25830627 10.1371/journal.ppat.1004800 PMC4382300

[mpp13354-bib-0022] Dodd, A.N. , Kudla, J. & Sanders, D. (2010) The language of calcium signaling. Annual Review of Plant Biology, 61, 593–620. 10.1146/annurev-arplant-070109-104628 20192754

[mpp13354-bib-0023] Dubey, A. , Barad, S. , Luria, N. , Kumar, D. , Espeso, E.A. & Prusky, D. (2016) Cation‐stress‐responsive transcription factors SltA and CrzA regulate morphogenetic processes and pathogenicity of *Colletotrichum gloeosporioides* . PLoS One, 11, e0168561. 28030573 10.1371/journal.pone.0168561 PMC5193415

[mpp13354-bib-0024] Fang, W. , Yang, L. , Zhu, X. , Zeng, L. & Li, X. (2013) Seasonal and habitat dependent variations in culturable endophytes of *Camellia sinensis* . Journal of Plant Pathology & Microbiology, 4, 1000169.

[mpp13354-bib-0025] Fang, X. , Chai, W. , Li, S. , Zhang, L. , Yu, H. , Shen, J. et al. (2021) HSP17.4 mediates salicylic acid and jasmonic acid pathways in the regulation of resistance to *Colletotrichum gloeosporioides* in strawberry. Molecular Plant Pathology, 22, 817–828. 33951267 10.1111/mpp.13065 PMC8232031

[mpp13354-bib-0026] Fire, A. , Xu, S. , Montgomery, M.K. , Kostas, S. , Driver, S.E. & Mello, C.C. (1998) Potent and specific genetic interference by double‐stranded RNA in *Caenorhabditis elegans* . Nature, 391, 806–811. 9486653 10.1038/35888

[mpp13354-bib-0027] Fujita, M. , Fujita, Y. , Noutoshi, Y. , Takahashi, F. , Narusaka, Y. , Yamaguchi‐Shinozaki, K. et al. (2006) Crosstalk between abiotic and biotic stress responses: a current view from the points of convergence in the stress signaling networks. Current Opinion in Plant Biology, 9, 436–442. 16759898 10.1016/j.pbi.2006.05.014

[mpp13354-bib-0028] Gao, X. , Wang, Q. , Feng, Q. , Zhang, B. , He, C. , Luo, H. et al. (2022) Heat shock transcription factor CgHSF1 is required for melanin biosynthesis, appressorium formation, and pathogenicity in *Colletotrichum gloeosporioides* . Journal of Fungi, 8, 175. 35205929 10.3390/jof8020175 PMC8876323

[mpp13354-bib-0029] George Thompson, A.M. , Iancu, C.V. , Neet, K.E. , Dean, J.V. & Choe, J. (2017) Differences in salicylic acid glucose conjugations by UGT74F1 and UGT74F2 from *Arabidopsis thaliana* . Scientific Reports, 7, 46629. 28425481 10.1038/srep46629 PMC5397973

[mpp13354-bib-0030] Göhre, V. , Jones, A.M. , Sklenar, J. , Robatzek, S. & Weber, A.P. (2012) Molecular crosstalk between PAMP‐triggered immunity and photosynthesis. Molecular Plant‐Microbe Interactions, 25, 1083–1092. 22550958 10.1094/MPMI-11-11-0301

[mpp13354-bib-0031] Goodwin, P.H. & Chen, G.Y. (2002) High expression of a sucrose non‐fermenting (SNF1)‐related protein kinase from *Colletotrichum gloeosporoides* f. sp. *malvae* is associated with penetration of *Malva pusilla* . FEMS Microbiology Letters, 215, 169–174. 12399031 10.1111/j.1574-6968.2002.tb11387.x

[mpp13354-bib-0032] Goring, D.R. & Walker, J.C. (2004) Self‐rejection–a new kinase connection. Science, 303, 1474–1475. 15001763 10.1126/science.1095764

[mpp13354-bib-0033] Gorshkov, V.Y. & Tsers, I.D. (2021) Plant susceptible responses: the underestimated side of plant–pathogen interactions. Biological Reviews of the Cambridge Philosophical Society, 97, 45–66. 34435443 10.1111/brv.12789 PMC9291929

[mpp13354-bib-0034] Guo, M. , Pan, Y.M. , Dai, Y.L. & Gao, Z.M. (2014) First report of brown blight disease caused by *Colletotrichum gloeosporioides* on *Camellia sinensis* in Anhui province, China. Plant Disease, 98, 284. 10.1094/PDIS-08-13-0896-PDN 30708777

[mpp13354-bib-0035] He, P. , Wang, Y. , Wang, X. , Zhang, X. & Tian, C. (2017) The mitogen‐activated protein kinase CgMK1 governs appressorium formation, melanin synthesis, and plant infection of *Colletotrichum gloeosporioides* . Frontiers in Microbiology, 8, 2216. 29176970 10.3389/fmicb.2017.02216 PMC5686099

[mpp13354-bib-0036] Hentrich, M. , Böttcher, C. , Düchting, P. , Cheng, Y. , Zhao, Y. , Berkowitz, O. et al. (2013) The jasmonic acid signaling pathway is linked to auxin homeostasis through the modulation of YUCCA8 and YUCCA9 gene expression. The Plant Journal, 74, 626–637. 23425284 10.1111/tpj.12152 PMC3654092

[mpp13354-bib-0037] Houben, M. & Van de Poel, B. (2019) 1‐aminocyclopropane‐1‐carboxylic acid oxidase (ACO): the enzyme that makes the plant hormone ethylene. Frontiers in Plant Science, 10, 695. 31191592 10.3389/fpls.2019.00695 PMC6549523

[mpp13354-bib-0038] Howard, R.J. & Ferrari, M.A. (1989) Role of melanin in appressorium function. Experimental Mycology, 13, 403–418.

[mpp13354-bib-0135] Hu, G. , Wei, K. , Zhang, Y. , Bao, W. & Liang, D. (2021). Estimation of tea leaf blight severity in natural scene images. Precision Agriculture, 22, 1239–1262.

[mpp13354-bib-0039] Hwang, C.S. , Flaishman, M.A. & Kolattukudy, P.E. (1995) Cloning of a gene expressed during appressorium formation by *Colletotrichum gloeosporioides* and a marked decrease in virulence by disruption of this gene. The Plant Cell, 7, 183–193. 7756829 10.1105/tpc.7.2.183 PMC160774

[mpp13354-bib-0040] Ibraheem, F. , Gaffoor, I. , Tan, Q. , Shyu, C. & Chopra, S. (2015) A sorghum MYB transcription factor induces 3‐deoxyanthocyanidins and enhances resistance against leaf blights in maize. Molecules, 20, 2388–2404. 25647576 10.3390/molecules20022388 PMC6272393

[mpp13354-bib-0041] Jeyaraj, A. , Chandran, V. & Gajjeraman, P. (2014) Differential expression of microRNAs in dormant bud of tea [*Camellia sinensis* (L.) O. Kuntze]. Plant Cell Reports, 33, 1053–1069. 24658841 10.1007/s00299-014-1589-4

[mpp13354-bib-0042] Jeyaraj, A. , Elango, T. , Li, X. & Guo, G. (2020) Utilization of microRNAs and their regulatory functions for improving biotic stress tolerance in tea plant [*Camellia sinensis* (L.) O. Kuntze]. RNA Biology, 17, 1365–1382. 32478595 10.1080/15476286.2020.1774987 PMC7549669

[mpp13354-bib-0043] Jeyaraj, A. , Elango, T. , Yu, Y. , Chen, X. , Zou, Z. , Ding, Z. et al. (2021) Impact of exogenous caffeine on regulatory networks of microRNAs in response to *Colletotrichum gloeosporioides* in tea plant. Scientia Horticulturae, 279, 109914.

[mpp13354-bib-0044] Jeyaraj, A. , Wang, X. , Wang, S. , Liu, S. , Zhang, R. , Wu, A. et al. (2019) Identification of regulatory networks of MicroRNAs and their targets in response to *Colletotrichum gloeosporioides* in tea plant (*Camellia sinensis* L.). Frontiers in Plant Science, 10, 1096. 31572415 10.3389/fpls.2019.01096 PMC6751461

[mpp13354-bib-0045] Jiang, L. , Wu, P. , Yang, L. , Liu, C. , Guo, P. , Wang, H. et al. (2021) Transcriptomics and metabolomics reveal the induction of flavonoid biosynthesis pathway in the interaction of *Stylosanthes*–*Colletotrichum gloeosporioides* . Genomics, 113, 2702–2716. 34111523 10.1016/j.ygeno.2021.06.004

[mpp13354-bib-0046] Jones, J.D. & Dangl, J.L. (2006) The plant immune system. Nature, 444, 323–329. 17108957 10.1038/nature05286

[mpp13354-bib-0047] Jwa, N. & Hwang, B.K. (2017) Convergent evolution of pathogen effectors toward reactive oxygen species signaling networks in plants. Frontiers in Plant Science, 8, 1687. 29033963 10.3389/fpls.2017.01687 PMC5627460

[mpp13354-bib-0048] Kato, M. , Mizuno, K. , Crozier, A. , Fujimura, T. & Ashihara, H. (2000) Plant biotechnology: caffeine synthase gene from tea leaves. Nature, 406, 956–957. 10984041 10.1038/35023072

[mpp13354-bib-0049] Kazan, K. & Manners, J. (2013) MYC2: the master in action. Molecular Plant, 6, 686–703. 23142764 10.1093/mp/sss128

[mpp13354-bib-0050] Kim, Y. , Kawano, T. , Li, D. & Kolattukudy, P.E. (2000) A mitogen‐activated protein kinase kinase required for induction of cytokinesis and appressorium formation by host signals in the conidia of *Colletotrichum gloeosporioides* . The Plant Cell, 12, 1331–1343. 10948253 10.1105/tpc.12.8.1331 PMC149106

[mpp13354-bib-0051] Kim, Y. , Wang, Y. , Liu, Z. & Kolattukudy, P.E. (2002) Identification of a hard surface contact‐induced gene in *Colletotrichum gloeosporioides* conidia as a sterol glycosyl transferase, a novel fungal virulence factor. The Plant Journal, 30, 177–187. 12000454 10.1046/j.1365-313x.2002.01284.x

[mpp13354-bib-0052] Kobayashi, M. , Ohura, I. , Kawakita, K. , Yokota, N. , Fujiwara, M. , Shimamoto, K. et al. (2007) Calcium‐dependent protein kinases regulate the production of reactive oxygen species by potato NADPH oxidase. The Plant Cell, 19, 1065–1080. 17400895 10.1105/tpc.106.048884 PMC1867354

[mpp13354-bib-0053] Koch, A. & Kogel, K. (2014) New wind in the sails: improving the agronomic value of crop plants through RNAi‐mediated gene silencing. Plant Biotechnology Journal, 12, 821–831. 25040343 10.1111/pbi.12226

[mpp13354-bib-0054] Koornneef, A. & Pieterse, C.M. (2008) Cross talk in defense signaling. Plant Physiology, 146, 839–844. 18316638 10.1104/pp.107.112029 PMC2259093

[mpp13354-bib-0055] Ku, Y. , Sintaha, M. , Cheung, M. & Lam, H. (2018) Plant hormone signaling crosstalks between biotic and abiotic stress responses. International Journal of Molecular Sciences, 19, 3206. 30336563 10.3390/ijms19103206 PMC6214094

[mpp13354-bib-0056] Kumar, V. , Gupta, V.P. , Babu, A.M. , Mishra, R.K. , Thiagarajan, V. & Datta, R.K. (2001) Surface ultrastructural studies on penetration and infection process of *Colletotrichum gloeosporioides* on mulberry leaf causing black spot disease. Journal of Phytopathology, 149, 629–633.

[mpp13354-bib-0057] Langfelder, K. , Streibel, M. , Jahn, B. , Haase, G. & Brakhage, A.A. (2003) Biosynthesis of fungal melanins and their importance for human pathogenic fungi. Fungal Genetics and Biology, 38, 143–158. 12620252 10.1016/s1087-1845(02)00526-1

[mpp13354-bib-0058] Leon‐Reyes, A. , Van der Does, D. , De Lange, E.S. , Delker, C. , Wasternack, C. , Van Wees, S.C. et al. (2010) Salicylate‐mediated suppression of jasmonate‐responsive gene expression in Arabidopsis is targeted downstream of the jasmonate biosynthesis pathway. Planta, 232, 1423–1432. 20839007 10.1007/s00425-010-1265-z PMC2957573

[mpp13354-bib-0059] Li, X. , Ahammed, G.J. , Li, Z. , Tang, M. , Yan, P. & Han, W. (2016) Decreased biosynthesis of jasmonic acid via lipoxygenase pathway compromised caffeine‐induced resistance to *Colletotrichum gloeosporioides* under elevated CO_2_ in tea seedlings. Phytopathology, 106, 1270–1277. 27392179 10.1094/PHYTO-12-15-0336-R

[mpp13354-bib-0060] Li, X. , Ke, Z. , Yu, X. , Liu, Z. & Zhang, C. (2019) Transcription factor CgAzf1 regulates melanin production, conidial development and infection in *Colletotrichum gloeosporioides* . Antonie Van Leeuwenhoek, 112, 1095–1104. 30725325 10.1007/s10482-019-01243-1

[mpp13354-bib-0061] Li, X. , Wu, Y. , Liu, Z. & Zhang, C. (2017) The function and transcriptome analysis of a bZIP transcription factor CgAP1 in *Colletotrichum gloeosporioides* . Microbiological Research, 197, 39–48. 28219524 10.1016/j.micres.2017.01.006

[mpp13354-bib-0062] Liang, C. , Zhang, B. , Zhou, Y. , Yin, H. , An, B. , Lin, D. et al. (2021) CgNPG1 as a novel pathogenic gene of *Colletotrichum gloeosporioides* from *Hevea brasiliensis* in mycelial growth, conidiation, and the invasive structures development. Frontiers in Microbiology, 12, 629387. 33763047 10.3389/fmicb.2021.629387 PMC7982478

[mpp13354-bib-0063] Lin, C. , Liu, X. , Shi, T. , Li, C. & Huang, G. (2018) The *Colletotrichum gloeosporioides* perilipin homologue CAP 20 regulates functional appressorial formation and fungal virulence. Journal of Phytopathology, 166, 216–225.

[mpp13354-bib-0064] Liu, F. , Weir, B.S. , Damm, U. , Crous, P.W. , Wang, Y. , Liu, B. et al. (2015) Unravelling *Colletotrichum* species associated with *Camellia*: employing *ApMat* and *GS* loci to resolve species in the *C. gloeosporioides* complex. Persoonia, 35, 63–86. 26823629 10.3767/003158515X687597 PMC4713112

[mpp13354-bib-0065] Liu, N. , Wang, Q. , He, C. & An, B. (2021) CgMFS1, a major facilitator superfamily transporter, is required for sugar transport, oxidative stress resistance, and pathogenicity of *Colletotrichum gloeosporioides* from *Hevea brasiliensis* . Current Issues in Molecular Biology, 43, 1548–1557. 34698108 10.3390/cimb43030109 PMC8929089

[mpp13354-bib-0066] Loon, L.C. & Strien, E.V. (1999) The families of pathogenesis‐related proteins, their activities, and comparative analysis of PR‐1 type proteins. Physiological and Molecular Plant Pathology, 55, 85–97.

[mpp13354-bib-0067] Lorence, A. & Verpoorte, R. (2004) Gene transfer and expression in plants. Methods in Molecular Biology, 267, 329–350. 15269435 10.1385/1-59259-774-2:329

[mpp13354-bib-0068] Lu, Q. , Wang, Y. , Li, N. , Ni, D. , Yang, Y. & Wang, X. (2018) Differences in the characteristics and pathogenicity of *Colletotrichum camelliae* and *C. fructicola* isolated from the tea plant [*Camellia sinensis* (L.) O. Kuntze]. Frontiers in Microbiology, 9, 3060. 30619146 10.3389/fmicb.2018.03060 PMC6297754

[mpp13354-bib-0069] Lu, Q. , Wang, Y. , Xiong, F. , Hao, X. , Zhang, X. , Li, N. et al. (2020) Integrated transcriptomic and metabolomic analyses reveal the effects of callose deposition and multihormone signal transduction pathways on the tea plant–*Colletotrichum camelliae* interaction. Scientific Reports, 10, 12858. 32733080 10.1038/s41598-020-69729-x PMC7393116

[mpp13354-bib-0070] Ma, W. & Berkowitz, G.A. (2011) Ca^2+^ conduction by plant cyclic nucleotide gated channels and associated signaling components in pathogen defense signal transduction cascades. New Phytologist, 190, 566–572. 21166809 10.1111/j.1469-8137.2010.03577.x

[mpp13354-bib-0071] Miyara, I. , Shnaiderman, C. , Meng, X. , Vargas, W.A. , Díaz‐Mínguez, J.M. , Sherman, A. et al. (2012) Role of nitrogen‐metabolism genes expressed during pathogenicity of the alkalinizing *Colletotrichum gloeosporioides* and their differential expression in acidifying pathogens. Molecular Plant‐Microbe Interactions, 25, 1251–1263. 22571816 10.1094/MPMI-01-12-0017-R

[mpp13354-bib-0072] Morin, L. , Derby, J. & Kokko, E.G. (1996) Infection process of *Colletotrichum gloeosporioides* f. sp. *malvae* on Malvaceae weeds. Fungal Biology, 100, 165–172.

[mpp13354-bib-0073] Mukhopadhyay, M. , Mondal, T.K. & Chand, P.K. (2016) Biotechnological advances in tea (*Camellia sinensis* [L.] O. Kuntze): a review. Plant Cell Reports, 35, 255–287. 26563347 10.1007/s00299-015-1884-8

[mpp13354-bib-0074] Müller, M. & Munné‐Bosch, S. (2015) Ethylene response factors: a key regulatory hub in hormone and stress signaling. Plant Physiology, 169, 32–41. 26103991 10.1104/pp.15.00677 PMC4577411

[mpp13354-bib-0075] Münch, S. , Lingner, U. , Floss, D.S. , Ludwig, N. , Sauer, N. & Deising, H.B. (2008) The hemibiotrophic lifestyle of *Colletotrichum* species. Journal of Plant Physiology, 165, 41–51. 17765357 10.1016/j.jplph.2007.06.008

[mpp13354-bib-0076] Mur, L.A. , Kenton, P. , Atzorn, R. , Miersch, O. & Wasternack, C. (2006) The outcomes of concentration‐specific interactions between salicylate and jasmonate signaling include synergy, antagonism, and oxidative stress leading to cell death. Plant Physiology, 140, 249–262. 16377744 10.1104/pp.105.072348 PMC1326048

[mpp13354-bib-0077] Musiał, C. , Kuban‐Jankowska, A. & Górska‐Ponikowska, M. (2020) Beneficial properties of green tea catechins. International Journal of Molecular Sciences, 21, 1744. 32143309 10.3390/ijms21051744 PMC7084675

[mpp13354-bib-0078] Naseem, M. , Kaltdorf, M. & Dandekar, T. (2015) The nexus between growth and defence signalling: auxin and cytokinin modulate plant immune response pathways. Journal of Experimental Botany, 66, 4885–4896. 26109575 10.1093/jxb/erv297

[mpp13354-bib-0079] Nesher, I. , Minz, A. , Kokkelink, L. , Tudzynski, P. & Sharon, A. (2011) Regulation of pathogenic spore germination by CgRac1 in the fungal plant pathogen *Colletotrichum gloeosporioides* . Eukaryotic Cell, 10, 1122–1130. 21460190 10.1128/EC.00321-10 PMC3165446

[mpp13354-bib-0080] Nowara, D. , Gay, A. , Lacomme, C. , Shaw, J. , Ridout, C.J. , Douchkov, D. et al. (2010) HIGS: host‐induced gene silencing in the obligate biotrophic fungal pathogen *Blumeria graminis* . The Plant Cell, 22, 3130–3141. 20884801 10.1105/tpc.110.077040 PMC2965548

[mpp13354-bib-0081] Nwankiti, A.O. , Okoli, O.O. & Okpala, E.U. (1987) Screening of water yam (*Dioscorea alata*) cultivars for tolerance to anthracnose/blotch disease. Fitopatologia Brasileira, 12, 36–39.

[mpp13354-bib-0082] Oren, M. , Hudgell, M.A. , Golconda, P. , Lun, C.M. & Smith, L.C. (2016) Genomic instability and shared mechanisms for gene diversification in two distant immune gene families: the plant NBS‐LRR genes and the echinoid 185/333 genes. In: Malagoli, D. (Ed.) The evolution of the immune system: conservation and diversification. London, UK: Elsevier‐Academic Press, pp. 295–310.

[mpp13354-bib-0083] Pan, Y. , Li, L. , Yang, J. , Li, B. , Zhang, Y. , Wang, P. et al. (2022) Involvement of protein kinase CgSat4 in potassium uptake, cation tolerance, and full virulence in *Colletotrichum gloeosporioides* . Frontiers in Plant Science, 13, 773898. 35463420 10.3389/fpls.2022.773898 PMC9021643

[mpp13354-bib-0084] Pandey, A.K. , Burlakoti, R.R. , Kenyon, L. & Nair, R.M. (2018) Perspectives and challenges for sustainable management of fungal diseases of mungbean [*Vigna radiata* (L.) R. Wilczek var. *radiata*]: a review. Frontiers in Environmental Science, 6, 53.

[mpp13354-bib-0085] Park, S. , Kaimoyo, E. , Kumar, D. , Mosher, S. & Klessig, D.F. (2007) Methyl salicylate is a critical mobile signal for plant systemic acquired resistance. Science, 318, 113–116. 17916738 10.1126/science.1147113

[mpp13354-bib-0086] Perfect, S.E. , Hughes, H.B. , O'Connell, R.J. & Green, J.R. (1999) *Colletotrichum*: a model genus for studies on pathology and fungal–plant interactions. Fungal Genetics and Biology, 27, 186–198. 10441444 10.1006/fgbi.1999.1143

[mpp13354-bib-0087] Pratyusha, D.S. & Sarada, D.V. (2022) MYB transcription factors‐master regulators of phenylpropanoid biosynthesis and diverse developmental and stress responses. Plant Cell Reports, 41, 2245–2260. 36171500 10.1007/s00299-022-02927-1

[mpp13354-bib-0088] Price, D.R. & Gatehouse, J.A. (2008) RNAi‐mediated crop protection against insects. Trends in Biotechnology, 26, 393–400. 18501983 10.1016/j.tibtech.2008.04.004

[mpp13354-bib-0089] Rabha, A.J. , Naglot, A. , Sharma, G.D. , Gogoi, H.K. & Veer, V.S. (2014) In vitro evaluation of antagonism of endophytic *Colletotrichum gloeosporioides* against potent fungal pathogens of *Camellia sinensis* . Indian Journal of Microbiology, 54, 302–309. 24891737 10.1007/s12088-014-0458-8 PMC4039731

[mpp13354-bib-0090] Ross, J.R. , Nam, K.H. , D'Auria, J.C. & Pichersky, E. (1999) *S*‐adenosyl‐L‐methionine:salicylic acid carboxyl methyltransferase, an enzyme involved in floral scent production and plant defense, represents a new class of plant methyltransferases. Archives of Biochemistry and Biophysics, 367, 9–16. 10375393 10.1006/abbi.1999.1255

[mpp13354-bib-0091] Ruan, J. , Zhou, Y. , Zhou, M. , Yan, J. , Khurshid, M. , Weng, W. et al. (2019) Jasmonic acid signaling pathway in plants. International Journal of Molecular Sciences, 20, 2479. 31137463 10.3390/ijms20102479 PMC6566436

[mpp13354-bib-0092] Saijo, Y. , Loo, E.P. & Yasuda, S. (2018) Pattern recognition receptors and signaling in plant–microbe interactions. The Plant Journal, 93, 592–613. 29266555 10.1111/tpj.13808

[mpp13354-bib-0093] Shan, W. , Chen, J. , Kuang, J. & Lu, W. (2016) Banana fruit NAC transcription factor MaNAC5 cooperates with MaWRKYs to enhance the expression of pathogenesis‐related genes against *Colletotrichum musae* . Molecular Plant Pathology, 17, 330–338. 26033522 10.1111/mpp.12281 PMC6638545

[mpp13354-bib-0094] Sharma, M. & Kulshrestha, S. (2015) *Colletotrichum gloeosporioides*: an anthracnose causing pathogen of fruits and vegetables. Biosciences Biotechnology Research Asia, 12, 1233–1246.

[mpp13354-bib-0095] Shi, N. , Du, Y. , Ruan, H. , Yang, X. , Dai, Y. , Gan, L. et al. (2018) First report of *Colletotrichum fructicola* causing anthracnose on *Camellia sinensis* in Guangdong province, China. Plant Disease, 102, 241.

[mpp13354-bib-0136] Shi, Y.‐L. , Sheng, Y.‐Y. , Cai, Z.‐Y. , Yang, R. , Li, Q.‐S. , Li, X.‐M. et al. (2019). Involvement of salicylic acid in anthracnose infection in tea plants revealed by transcriptome profiling. International Journal of Molecular Sciences, 20, 2439. 31108845 10.3390/ijms20102439 PMC6566613

[mpp13354-bib-0096] Shih, J. , Wei, Y. & Goodwin, P.H. (2000) A comparison of the pectate lyase genes, *pel‐1* and *pel‐2*, of *Colletotrichum gloeosporioides* f.sp. *malvae* and the relationship between their expression in culture and during necrotrophic infection. Gene, 243, 139–150. 10675622 10.1016/s0378-1119(99)00546-6

[mpp13354-bib-0097] Silva, D.D. , Crous, P.W. , Ades, P.K. , Hyde, K.D. & Taylor, P.W. (2017) Life styles of *Colletotrichum* species and implications for plant biosecurity. Fungal Biology Reviews, 31, 155–168.

[mpp13354-bib-0098] Sinha, A.K. , Jaggi, M. , Raghuram, B. & Tuteja, N.K. (2011) Mitogen‐activated protein kinase signaling in plants under abiotic stress. Plant Signaling & Behavior, 6, 196–203. 21512321 10.4161/psb.6.2.14701 PMC3121978

[mpp13354-bib-0099] Song, S. , Huang, H. , Gao, H. , Wang, J. , Wu, D. , Liu, X. et al. (2014) Interaction between MYC2 and ETHYLENE INSENSITIVE3 modulates antagonism between jasmonate and ethylene signaling in *Arabidopsis* . The Plant Cell, 26, 263–279. 24399301 10.1105/tpc.113.120394 PMC3963574

[mpp13354-bib-0100] Song, X. , Gu, K. , Duan, X. , Xiao, X. , Hou, Y. , Duan, Y. et al. (2018) A myosin5 dsRNA that reduces the fungicide resistance and pathogenicity of *Fusarium asiaticum* . Pesticide Biochemistry and Physiology, 150, 1–9. 30195381 10.1016/j.pestbp.2018.07.004

[mpp13354-bib-0101] Spanu, P.D. & Panstruga, R. (2017) Editorial: biotrophic plant–microbe interactions. Frontiers in Plant Science, 8, 192. 28243250 10.3389/fpls.2017.00192 PMC5303711

[mpp13354-bib-0102] Spoel, S.H. & Dong, X. (2008) Making sense of hormone crosstalk during plant immune responses. Cell Host & Microbe, 3, 348–351. 18541211 10.1016/j.chom.2008.05.009

[mpp13354-bib-0103] Spoel, S.H. , Johnson, J.S. & Dong, X. (2007) Regulation of tradeoffs between plant defenses against pathogens with different lifestyles. Proceedings of the National Academy of Sciences of the United States of America, 104, 18842–18847. 17998535 10.1073/pnas.0708139104 PMC2141864

[mpp13354-bib-0104] Stephenson, S.A. , Stephens, C. , Maclean, D.J. & Manners, J. (2005) *CgDN24*: a gene involved in hyphal development in the fungal phytopathogen *Colletotrichum gloeosporioides* . Microbiological Research, 160, 389–397. 16255144 10.1016/j.micres.2005.03.003

[mpp13354-bib-0105] Stotz, H. , Mitrousia, G.K. , de Wit, P.J. & Fitt, B.D. (2014) Effector‐triggered defence against apoplastic fungal pathogens. Trends in Plant Science, 19, 491–500. 24856287 10.1016/j.tplants.2014.04.009 PMC4123193

[mpp13354-bib-0106] Sugiyama, A. , Sano, C.M. , Yazaki, K. & Sano, H. (2016) Caffeine fostering of mycoparasitic fungi against phytopathogens. Plant Signaling & Behavior, 11, e1113362. 26529400 10.1080/15592324.2015.1113362 PMC4871636

[mpp13354-bib-0107] Truman, W. , Bennett, M.H. , Kubigsteltig, I. , Turnbull, C. & Grant, M. (2007) *Arabidopsis* systemic immunity uses conserved defense signaling pathways and is mediated by jasmonates. Proceedings of the National Academy of Sciences of the United States of America, 104, 1075–1080. 17215350 10.1073/pnas.0605423104 PMC1783366

[mpp13354-bib-0108] Tsubouchi, H. , Terada, H. , Yamamoto, K. , Hisada, K. & Sakabe, Y. (1985) Caffeine degradation and increased ochratoxin A production by toxigenic strains of *Aspergillus ochraceus* isolated from green coffee beans. Mycopathologia, 90, 181–186. 4033738 10.1007/BF00436735

[mpp13354-bib-0109] Tsuda, K. & Katagiri, F. (2010) Comparing signaling mechanisms engaged in pattern‐triggered and effector‐triggered immunity. Current Opinion in Plant Biology, 13, 459–465. 20471306 10.1016/j.pbi.2010.04.006

[mpp13354-bib-0110] Tsuda, K. , Sato, M. , Stoddard, T.J. , Glazebrook, J. & Katagiri, F. (2009) Network properties of robust immunity in plants. PLoS Genetics, 5, e1000772. 20011122 10.1371/journal.pgen.1000772 PMC2782137

[mpp13354-bib-0111] Tugizimana, F. , Djami‐Tchatchou, A.T. , Steenkamp, P.A. , Piater, L.A. & Dubery, I.A. (2019) Metabolomic analysis of defense‐related reprogramming in *Sorghum bicolor* in response to *Colletotrichum sublineolum* infection reveals a functional metabolic web of phenylpropanoid and flavonoid pathways. Frontiers in Plant Science, 9, 1840. 30662445 10.3389/fpls.2018.01840 PMC6328496

[mpp13354-bib-0112] Vilela, B.J. , Pagés, M. & Lumbreras, V. (2010) Regulation of MAPK signaling and cell death by MAPK phosphatase MKP2. Plant Signaling & Behavior, 5, 1497–1500. 21057191 10.4161/psb.5.11.13645 PMC3115266

[mpp13354-bib-0113] Vitale, A. , Alfenas, A.C. , Siqueira, D.L. , Magistà, D. , Perrone, G. & Polizzi, G. (2020) Cultivar resistance against *Colletotrichum asianum* in the world collection of mango germplasm in southeastern Brazil. Plants, 9, 182. 32024312 10.3390/plants9020182 PMC7076395

[mpp13354-bib-0114] Wan, Y. , Zou, L. , Zeng, L. , Tong, H. & Chen, Y. (2021) A new *Colletotrichum* species associated with brown blight disease on *Camellia sinensis* . Plant Disease, 105, 1474–1481. 33258436 10.1094/PDIS-09-20-1912-RE

[mpp13354-bib-0115] Wang, M. & Dean, R.A. (2020) Movement of small RNAs in and between plants and fungi. Molecular Plant Pathology, 21, 589–601. 32027079 10.1111/mpp.12911 PMC7060135

[mpp13354-bib-0116] Wang, T. , Ren, D. , Guo, H. , Chen, X. , Zhu, P. , Nie, H. et al. (2020) CgSCD1 is essential for melanin biosynthesis and pathogenicity of *Colletotrichum gloeosporioides* . Pathogens, 9, 141. 32093195 10.3390/pathogens9020141 PMC7169410

[mpp13354-bib-0118] Wang, X. , Lu, D. & Tian, C. (2021a) CgEnd3 regulates endocytosis, appressorium formation, and virulence in the poplar anthracnose fungus *Colletotrichum gloeosporioides* . International Journal of Molecular Sciences, 22, 4029. 33919762 10.3390/ijms22084029 PMC8103510

[mpp13354-bib-0117] Wang, X. , Lu, D. & Tian, C. (2021b) Analysis of melanin biosynthesis in the plant pathogenic fungus *Colletotrichum gloeosporioides* . Fungal Biology, 125, 679–692. 34420695 10.1016/j.funbio.2021.04.004

[mpp13354-bib-0119] Wang, Y. , Chen, J. , Li, D. , Zheng, L. & Huang, J. (2016) CglCUT1 gene required for cutinase activity and pathogenicity of *Colletotrichum gloeosporioides* causing anthracnose of *Camellia oleifera* . European Journal of Plant Pathology, 147, 103–114.

[mpp13354-bib-0120] Wang, Y. , Hao, X. , Lu, Q. , Wang, L. , Qian, W. , Li, N. et al. (2018) Transcriptional analysis and histochemistry reveal that hypersensitive cell death and H_2_O_2_ have crucial roles in the resistance of tea plant (*Camellia sinensis* (L.) O. Kuntze) to anthracnose. Horticulture Research, 5, 18. 29619229 10.1038/s41438-018-0025-2 PMC5878829

[mpp13354-bib-0121] Wang, Y. , Hao, X. , Wang, L. , Xiao, B. , Wang, X. & Yang, Y. (2016) Diverse *Colletotrichum* species cause anthracnose of tea plants (*Camellia sinensis* (L.) O. Kuntze) in China. Scientific Reports, 6, 35287. 27782129 10.1038/srep35287 PMC5080629

[mpp13354-bib-0122] Wang, Y. , Wang, Z. , Yang, W. , Xie, X. , Cheng, H. , Qin, L. et al. (2019) Degradation of fungal microRNAs triggered by short tandem target mimics is via the small‐RNA‐degrading nuclease. Applied and Environmental Microbiology, 85, 9. 10.1128/AEM.03132-18 PMC6495759 30824452

[mpp13354-bib-0123] Wei, Y. , Shih, J. , Li, J. & Goodwin, P.H. (2002) Two pectin lyase genes, *pnl‐1* and *pnl‐2*, from *Colletotrichum gloeosporioides* f. sp. *malvae* differ in a cellulose‐binding domain and in their expression during infection of *Malva pusilla* . Microbiology, 148, 2149–2157. 12101302 10.1099/00221287-148-7-2149

[mpp13354-bib-0124] Weinberg, B. & Bealer, B.K. (2001) The world of caffeine: the science and culture of the world's most popular drug. London: Routledge.

[mpp13354-bib-0125] Werner, B.T. , Gaffar, F.Y. , Schuemann, J. , Biedenkopf, D. & Koch, A.M. (2020) RNA‐spray‐mediated silencing of *Fusarium graminearum* *AGO* and *DCL* genes improve barley disease resistance. Frontiers in Plant Science, 11, 476. 32411160 10.3389/fpls.2020.00476 PMC7202221

[mpp13354-bib-0126] Wilson, R.C. & Doudna, J.A. (2013) Molecular mechanisms of RNA interference. Annual Review of Biophysics, 42, 217–239. 10.1146/annurev-biophys-083012-130404 PMC5895182 23654304

[mpp13354-bib-0127] Yakoby, N. , Beno‐Moualem, D. , Keen, N.T. , Dinoor, A. , Pines, O. & Prusky, D. (2001) *Colletotrichum gloeosporioides* pelB is an important virulence factor in avocado fruit–fungus interaction. Molecular Plant‐Microbe Interactions, 14, 988–995. 11497471 10.1094/MPMI.2001.14.8.988

[mpp13354-bib-0128] Yang, Y. , Li, H. , Liu, M. , Wang, H. , Yang, Q. , Yan, D. et al. (2022) PeTGA1 enhances disease resistance against *Colletotrichum gloeosporioides* through directly regulating PeSARD1 in poplar. International Journal of Biological Macromolecules, 214, 672–684. 35738343 10.1016/j.ijbiomac.2022.06.099

[mpp13354-bib-0129] Zhang, L. , Li, X. , Zhou, Y. , Tan, G. & Zhang, L. (2021) Identification and characterization of *Colletotrichum* species associated with *Camellia sinensis* anthracnose in Anhui province, China. Plant Disease, 105, 2649–2657. 33342234 10.1094/PDIS-11-20-2335-RE

[mpp13354-bib-0130] Zhang, Y. , An, B. , Wang, W. , Zhang, B. , He, C. , Luo, H. et al. (2022) Actin‐bundling protein fimbrin regulates pathogenicity via organizing F‐Actin dynamics during appressorium development in *Colletotrichum gloeosporioides* . Molecular Plant Pathology, 23, 1472–1486. 35791045 10.1111/mpp.13242 PMC9452767

[mpp13354-bib-0131] Zheng, X. , Spivey, N.W. , Zeng, W. , Liu, P. , Fu, Z.Q. , Klessig, D.F. et al. (2012) Coronatine promotes *Pseudomonas syringae* virulence in plants by activating a signaling cascade that inhibits salicylic acid accumulation. Cell Host & Microbe, 11, 587–596. 22704619 10.1016/j.chom.2012.04.014 PMC3404825

[mpp13354-bib-0132] Zhu, Z. , An, F. , Feng, Y. , Li, P. , Xue, L. , Mu, A. et al. (2011) Derepression of ethylene‐stabilized transcription factors (EIN3/EIL1) mediates jasmonate and ethylene signaling synergy in *Arabidopsis* . Proceedings of the National Academy of Sciences of the United States of America, 108, 12539–12544. 21737749 10.1073/pnas.1103959108 PMC3145709

[mpp13354-bib-0133] Zipfel, C. (2014) Plant pattern‐recognition receptors. Trends in Immunology, 35, 345–351. 24946686 10.1016/j.it.2014.05.004

[mpp13354-bib-0134] Zotti, M.J. , dos Santos, E.Á. , Cagliari, D. , Christiaens, O. , Taning, C.N. & Smagghe, G. (2018) RNA interference technology in crop protection against arthropod pests, pathogens and nematodes. Pest Management Science, 74, 1239–1250. 29194942 10.1002/ps.4813

