# Supplementary figures and images for: Advances in understanding the mechanism of resistance to anthracnose and induced defence response in tea plants
Source: Mol Plant Pathol. 2023 Jul 31;24(10):1330–46. doi: 10.1111/mpp.13354 (PMC10502868; doi:10.1111/mpp.13354)

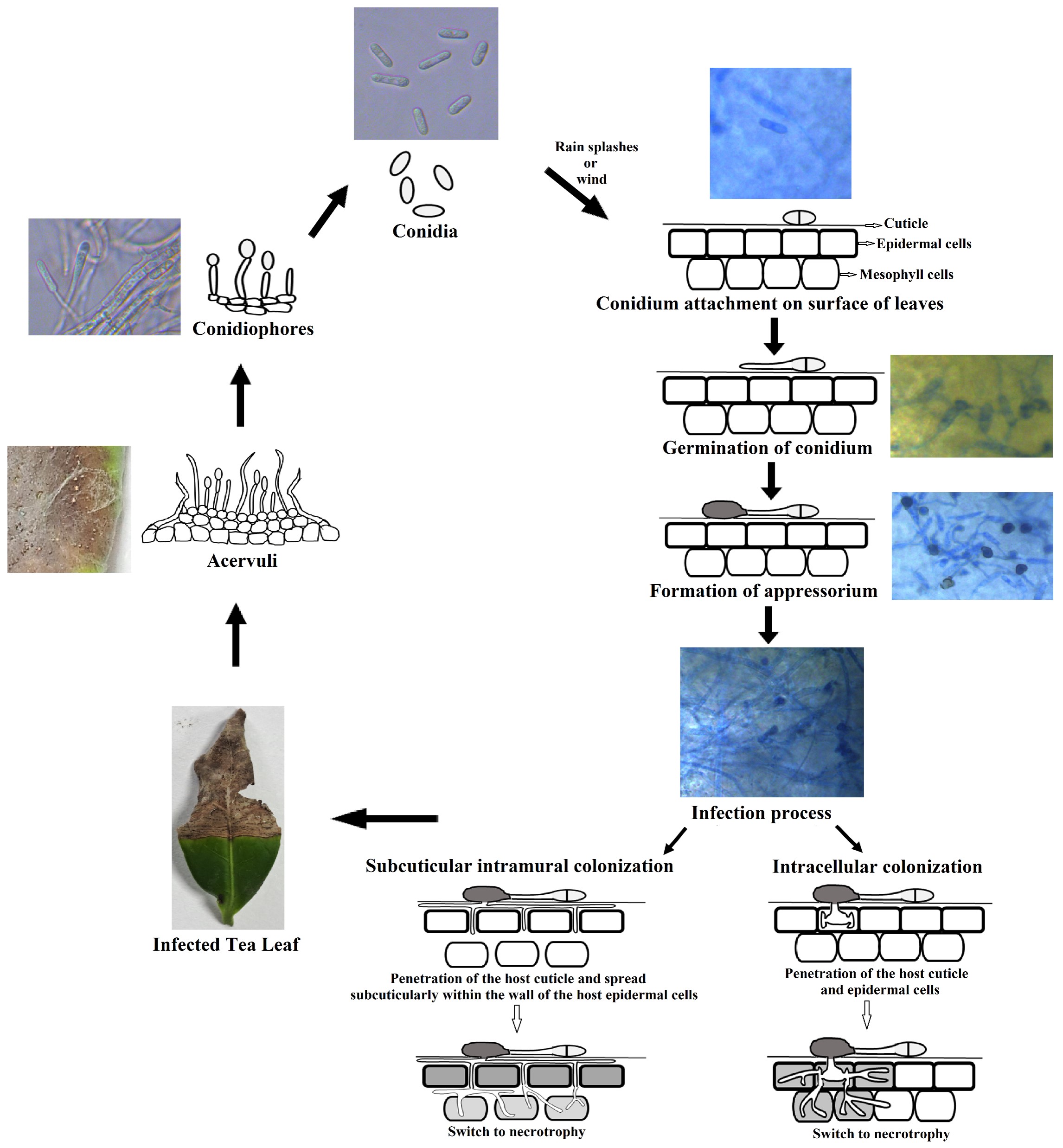

Supplement: Supplementary file 1 — FIGURE S1 Disease cycle of tea plant anthracnose caused by Colletotrichum spp. with microscope images displaying different stages of infection caused by the C. gloeosporioides on tea leaf. The conidia from necrotic lesions of infected leaves (acervuli) are dispersed by rain splashes or wind. After that, C. gloeosporioides can initiate different stages of infection, including conidium attachment on the surface of leaves, germination of conidium, formation of appressorium, and two postinfection strategies (intracellular hemibiotrophy and subcuticular intramural necrotrophy) for successful colonization of plant tissues. [file MPP-24-1330-s003.jpg]
